# Actuopaleoichnology of a modern Bay of Fundy macro-tidal flat: analogy with a Mississippian tidal flat deposit (Hartselle Sandstone) from Alabama

**DOI:** 10.7717/peerj.6975

**Published:** 2019-05-21

**Authors:** Louis G. Zachos, Brian F. Platt

**Affiliations:** 1 Institut für Evolutionsbiologie und Ökologie, Rheinische Friedrich-Wilhelms Universität Bonn, Bonn, Germany; 2 Department of Geology & Geological Engineering, University of Mississippi, University, MS, USA; 3 Center for Biodiversity & Conservation Research, University of Mississippi, University, MS, USA

**Keywords:** Ichnology, Littoral deposits, Trace fossils, Paleoecology, Invertebrates

## Abstract

Trace fossil zonation in the Hartselle Sandstone of Mississippian age (Chesterian: Visean-Serpukhovian) exposed on Fielder Ridge, Alabama is compared with modern macro-tidal flat ichnocoenoses on the Bay of Fundy at Lubec, Maine, and demonstrated to be analogous by sedimentologic and ichnotaxonomic criteria. The modern flat has minimal influence from either waves or freshwater influx, and can be divided into five distinct ichnocoenoses, characterized by surface traces (epichnia) and four sedimentologic facies defined by gross grain texture or hydrodynamic characteristics, but lacking significant surface traces. Several characteristics of tidal flat deposits in a fetch-limited, marine (i.e., non-estuarine), meso- to macro-tidal regime can be used to recognize similar environments as old as the late Paleozoic. These criteria include (1) limited influence of wind and waves on the depositional environment, (2) lack of significant freshwater influence and therefore any persistent brackish environments, (3) a distinct spatial distribution of microenvironments defined by substrate and exposure period, (4) high diversity of epichnial traces directly associated with microenvironments across the tidal flat, (5) generally low degree of reworking of traces by bioturbation but high degree of reworking by tidal currents, and (6) preservation of traces of predation and scavenging behavior on an exposed surface. These features, together with the regional depositional pattern of the Hartselle Sandstone interpreted as tide-influenced bars and shoals, support a meso- to macro-tidal interpretation of the depositional environment.

## Introduction

The height of tides and associated intertidal zones of the Bay of Fundy, bounded by the coasts of Maine, New Brunswick, and Nova Scotia, were noted by the great Scottish geologist Lyell (1845). Serious study of the sediments of the intertidal zone, reviewed by Amos (1984), came much later, when Klein (1963) delineated four depositional environments in the Bay of Fundy: wave-cut benches, estuarine clay flats, tidal flats in the lee of bedrock islands, and salt marshes. He further subdivided the tidal flats in the lee of bedrock islands, which are fetch-limited (protected from large waves), into a higher tidal flat characterized by gravel and sand, and a lower tidal flat with gravel, sand, silt, and clay.

Rocky shores were the subjects of some of the first studies of the ecological zonation of the intertidal zone (Benson, 2002), and these were soon followed by studies of sandy beaches and tidal flats (McLachlan, 1983). Newcombe (1935b) studied the physical environmental constraints of the intertidal zone at St. Andrews, New Brunswick, just across Passamaquoddy Bay from the current study area at Lubec, Maine. He recognized two “biomes” in the intertidal zone; one for the rocky shore and one for the sandy. Elmhirst (1932) was able to define zonation of intertidal areas of Scotland based on crustaceans (mostly amphipods) and Brady (1943) described and carefully quantified the infauna of sandy and muddy tidal flats on the Northumberland coast of England. Brady (1943) concluded that the zonation of the fauna was primarily due to the period of exposure and interspecific competition for food, although excessive silt or finer material had a negative impact on diversity and abundance. One of the first globally comprehensive studies of ecological zonation was by Dahl (1952), who divided the sandy beach environment into three vertically juxtaposed zones. He found that most of the organisms that leave the safety of their burrows and move across the exposed intertidal zone were crustaceans.

There is a long history of literature relating trace fossil assemblages to paleoenvironmental interpretation by analogy with neoichnological studies that can be traced back to the publications of James Dwight Dana, Thomas Rupert Jones, and John Walter Salter in the mid-1800s (Dawson, 1873). Tidal flats have been a substantial source of such studies (Frey, Howard & Hong, 1987; Mángano et al., 2002; Uchman & Pervesler, 2006), but there is less history for studies seeking to relate tidal regimes to particular ichnocoenoses (Gingras, MacEachern & Dashtgard, 2012). Many of these are focused on interpretation of traces as representative of organisms characteristic of tidal flats rather than the predictive value of such traces in the geologic record, with a few notable exceptions (Hertweck et al., 2005). A large number of these studies deal with areas in which there is significant mixing of fluvial-derived freshwater and marine water, and they focus on the distinction between brackish water and fully marine systems (Buatois et al., 2005; Hauck et al., 2009; Mángano & Buatois, 2004).

This study describes a modern macro-tidal environment with minimal wave or freshwater influence, thus limiting the complexities faced in other studies. The objective of the study is the definition of criteria used to recognize analogous tidal flat deposits in the geologic record. The targeted environments are meso- to macro-tidal regimes where waves and freshwater influx are minimal or non-existent. Emphasis is placed on ethological or behavioral aspects of modern tidal flat organisms as opposed to a strict ichnotaxonomic treatment. There are several classifications used to describe toponomy—the locations of traces relative to the substrate (Buatois & Mángano, 2011), and herein we use the system of Martinsson (1970) which includes epichnia (traces found on the top surface of strata), hypichnia (traces found on the bottom surface of strata), and endichnia (traces found wholly within a stratum). Because activity of the organisms at the interface between the substrate and the air is most characteristic of the exposed environment, ichnological zonation of the tidal flat is based on surface traces or epichnia. Grain size of sediment is important because of its relationship to oxygen content, permeability, and food availability (Dashtgard, Gingras & Pemberton, 2008). The direct effect of grain size on epifauna is minimal but these characteristics are important indirectly because the tidal flat fauna find refuge within the sediment when inundated. In a macro-tidal regime, benthic organisms are extremely active on the surfaces of exposed flats, but seek refuge in the substrate during inundation to avoid high-velocity tidal currents and predation (Reise, 1977; Warren, 1990). Epichnia in these settings, therefore, represent more diverse behaviors than endichnia. Significantly, in this study, intensive biological sampling in the modern field area allows identification of each trace maker with certainty. The results of the study of the modern tidal flat are compared with a Mississippian-age deposit for which epichnia are well preserved.

## Geographic and Geologic Settings

**Figure 1:**
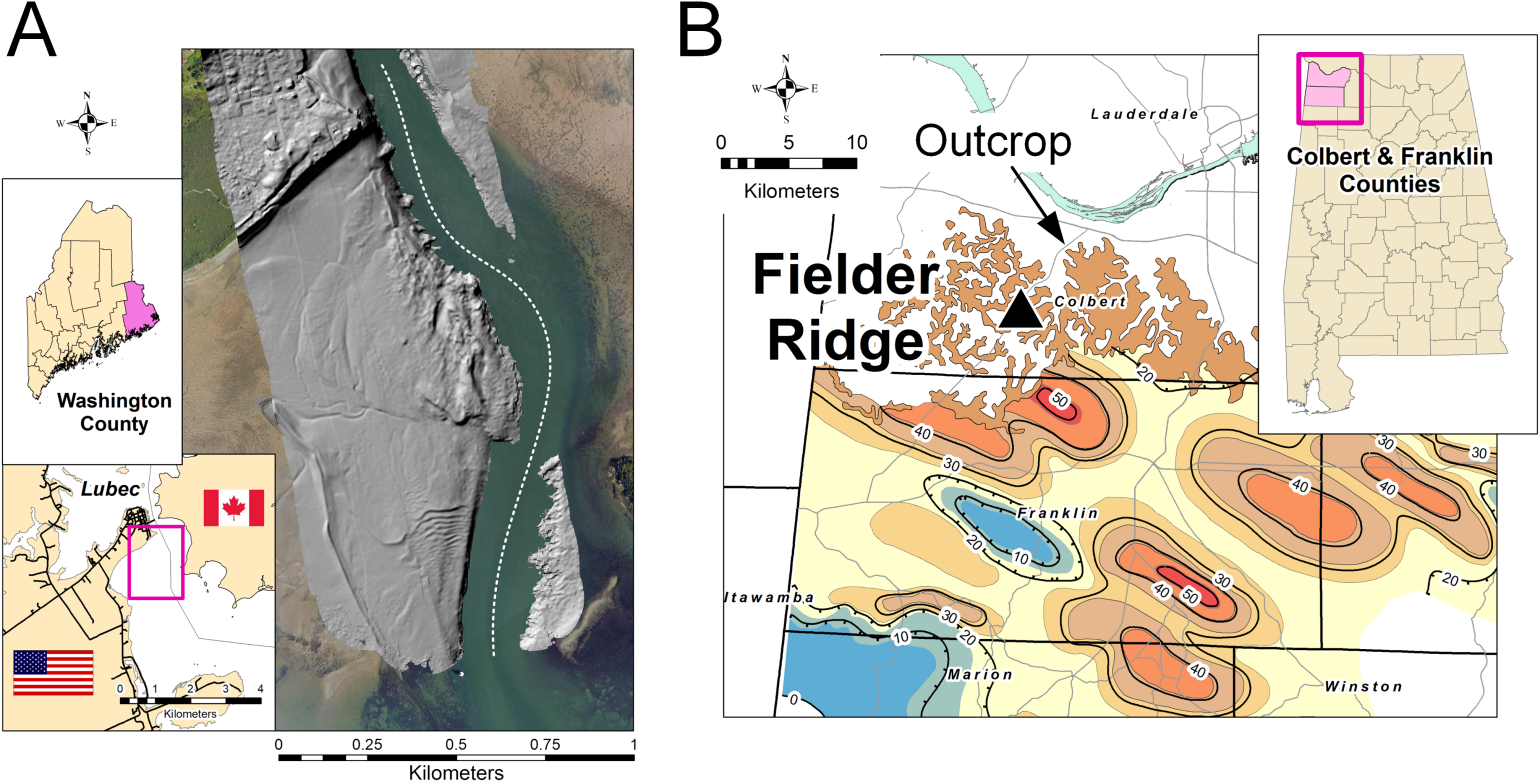
Study locations. (A) Modern tidal flat at Lubec, Washington County, Maine. Gray-scale hillshade image of Lidar displayed over aerial photograph to show area of interest. Dotted line shows approximate US/Canada border. (B) Hartselle Sandstone isopach map (contour interval 10 m) and outcrop extent, after Wilson (1987). Triangle marks locality at Fielder Ridge, Colbert County, Alabama.

The modern tidal flat is located at the United States—Canada border (latitude 44.853°, longitude −66.981°) near the town of Lubec, Maine (Fig. 1A), within the Lubec Embayment, which opens into the lower portion of the Bay of Fundy. The tidal flat extends for more than a kilometer from the beach at low tide. Tide ranges from six to seven m (Kelley, Belknap & Walsh, 2015), depending on lunar phase and time of year. The Lubec Embayment is a fetch-limited, partly enclosed bay that is protected from significant wave influence and has no appreciable sediment source other than coastal erosion (Walsh, 1988). Tectonic subsidence in the immediate area is nine mm/year (Lee & Diehl, 1989; Tyler & Ladd, 1980) and, with current rates of eustatic sea level rise, depositional accommodation exceeds one cm/year. The sediments are derived from the Pineo Ridge Moraine Complex glacial deposits (Gates, 1989; Kaplan, 2007) and these source deposits are heterogeneous in both grain size and mineralogy. The shoreline is backed by a freshwater marsh, which drains ephemerally though small streams or by shallow phreatic flow into the tidal flat, but otherwise there is no significant influx of freshwater and thus no brackish-water environment.

The Mississippian-age locality, a road cut exposing the Hartselle Sandstone and overlying Bangor Limestone, is a site in northwestern Alabama (latitude 34.648°, longitude −87.889°) called Fielder Ridge (Fig. 1B). The Hartselle Sandstone is a quartz arenite, Late Mississippian (Chesterian or Visean-Serpukhovian) in age (Pashin & Gastaldo, 2009). The Hartselle is exposed in northern Mississippi and Alabama in an east-west trending belt, extending southward in the subsurface, primarily within northwestern Alabama and restricted to the Black Warrior Basin (Wilson, 1987). The formation is underlain conformably by the Pride Mountain Formation, comprising shale and sandstone with minor limestone. It is overlain conformably by the Bangor Limestone, comprising limestone and shale. The Hartselle Sandstone has been interpreted as a barrier-bar system (Thomas, 1974), with associated back-barrier lagoons and barrier flats (Thomas & Mack, 1982). The Black Warrior Basin in the Late Mississippian was an enclosed embayment constrained to the east and northeast by the continental landmass resulting from the Appalachian orogeny, and to the south and southwest by the landmass formed by the Ouachita orogeny during this stage in the formation of Pangaea (Lacefield, 2013). Simulations have shown that the basin may have experienced meso- to macro-tidal fluxes during the Carboniferous (Wells et al., 2007), although with a slightly shorter period resulting from a shorter day (Williams, 2000). The northwest to southeast trend of the barrier bars, indicated by paleocurrent and isopach data (Thomas & Mack, 1982), is consistent with the existence of a strong tidal flux into and out of the Black Warrior Basin, opening to the northwest into the midcontinent epeiric sea. The Hartselle Sandstone is well exposed, although somewhat monotonous in character, within its outcrop belt. The unique exposure at Fielder Ridge, Colbert Co., Alabama, was first reported by Thomas (1972), who noted in particular the ripple-bedded section of interbedded shale and sandstone with abundant trace fossils. The ichnology of the Hartselle was intensively reviewed by Rindsberg (1994), with emphasis on collections from the Fielder Ridge outcrop.

## Materials and Methods

Observation and photography of modern surface traces of the Lubec ichnocoenoses were made over the period from 2008 to 2018, almost entirely during summer months. The actual invertebrate trace makers were collected in situ and identified, resulting in a direct association with respective trace suites. When possible, videos were made of organisms during trace making activities. Representative samples of the trace making organisms were preserved in ethanol. Slabs of ichnofossils were collected and recorded stratigraphically from Fielder Ridge, Alabama. Ichnofossils were whitened with ammonium chloride for photography using standard paleontological techniques (Feldmann, 1989; Sass, 1962). All figured ichnofossils are curated and housed in the collections of the Mississippi Museum of Natural Science (MMNS) in Jackson, Mississippi.

Descriptive terminology follows that of Trewin (1995) and Minter, Braddy & Davis (2007). In this study, the focus is on epichnial traces, although endichnial traces are considered where direct association with epichnial traces can be substantiated. Nearly all the ichnotaxonomic treatment of Mississippian ichnofossils follows Rindsberg (1994), with a few modifications as indicated in the text.

Identification of the modern fauna was greatly aided by a tabular key published by Pollock (1998), and supported with field guides (Watling, Fegley & Moring, 2003; Whitlach, 1982) and faunal lists (Larsen & Gilfillan, 2004; Trott, 2004).

For the modern tidal flat, detailed topobathymetric data and aerial photographs were obtained from a Lidar swath generated in 2014 by the New England District, U.S. Army Corps of Engineers Joint Airborne Lidar Bathymetry Technical Center of eXpertise and published by NOAA (2014) (https://coast.noaa.gov/htdata/lidar1_z/geoid12b/data/4911). These files were processed using ArcGIS® software. GIS files for Alabama were obtained from publicly available data published by the state (https://www.gsa.state.al.us).

Grain size distribution of the modern tidal flat sediment was determined using bulk sample dry sieving (Table S1). Sampling locations (Table S2) were recorded using GPS, and all spatial interpolation and contouring used ArcGIS®. Statistical treatment of results follows Folk (1980). Mean grain size is measured in phi (ø), where ø = −log_2_(*D*/*D*_0_) and *D* is the intermediate-axis grain diameter of ellipsoidal-shaped grains, rendered dimensionally consistent by division by the reference diameter *D*_0_ (equal to one mm). Sorting is the inclusive graphic standard deviation (the square root of the variance or second moment of the distribution). Skewness is a measure of the third moment or symmetry of the distribution, positive when fine-skewed, negative when coarse-skewed, and zero when symmetrical.

## Results

### Modern ichnocoenoses

**Figure 2:**
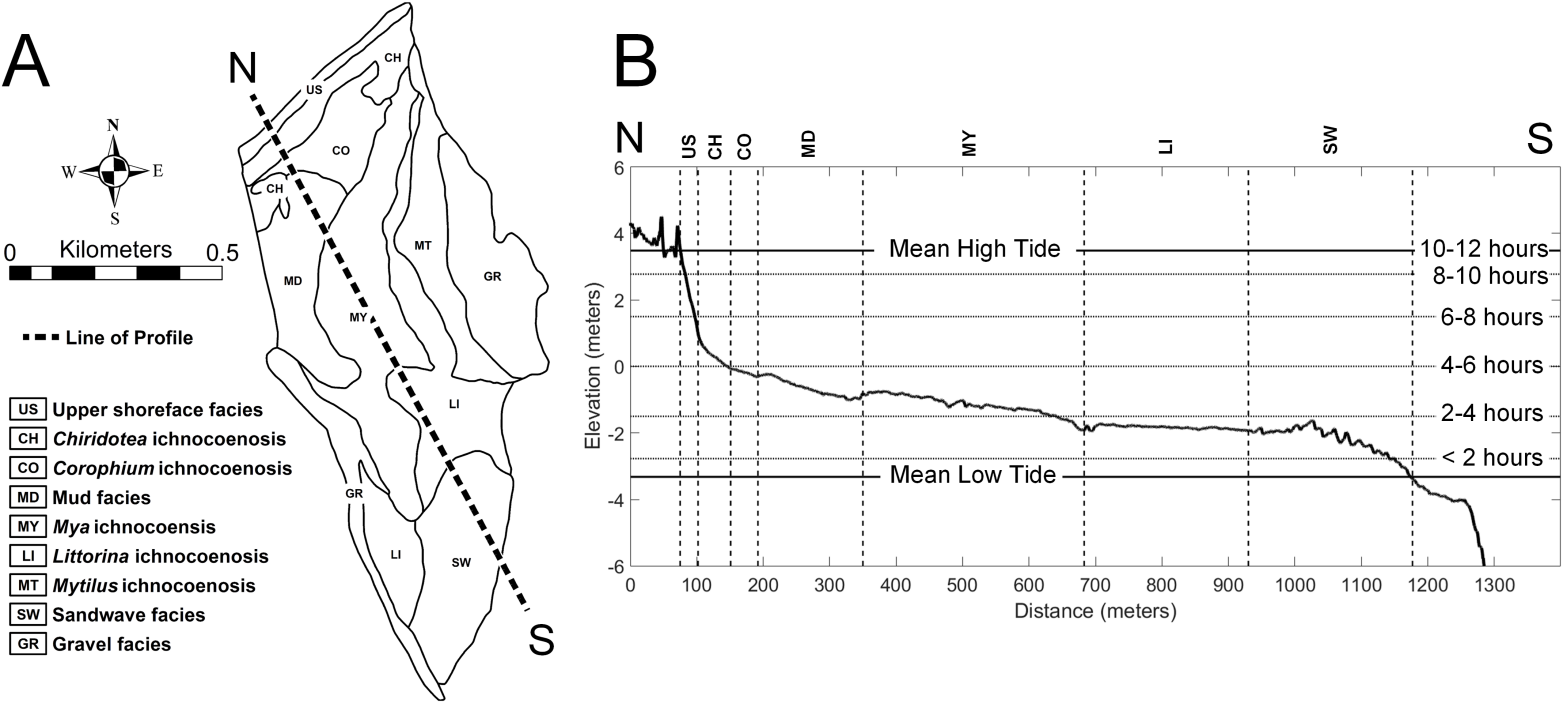
Topobathymetric profile of the modern tidal flat at Lubec, Maine, based on elevation derived from Lidar coverage. (A) Line of N-S profile with ichnocoenoses and facies delineation of tidal flat. (B) Profile from north (N) to south (S), vertical exaggeration 50:1. Horizontal lines mark exposure times during a tide cycle. Abbreviations: US, upper shoreface facies; CH, *Chiridotea* ichnocoenosis; CO, *Corophium* ichnocoenosis; MD, mud facies; MY, *Mya* ichnocoenosis; LI, *Littorina* ichnocoenosis; SW, sandwave facies.

The study area of approximately 60 ha on the tidal flat at Lubec, Maine, can be divided into a combined nine distinct facies and ichnocoenoses (Fig. 2A), all of which are described below. The elevation gradient increases monotonically toward the beach where the flat ends with a steep gradient to the mean high tide line (Fig. 2B). The surface of the flat is biologically heterogeneous with areas of the mid-flat covered with green and brown algae. The upper 10–15 cm of sediment are light-colored and oxic, with dark, H_2_S-rich sediment below.

**Figure 3:**
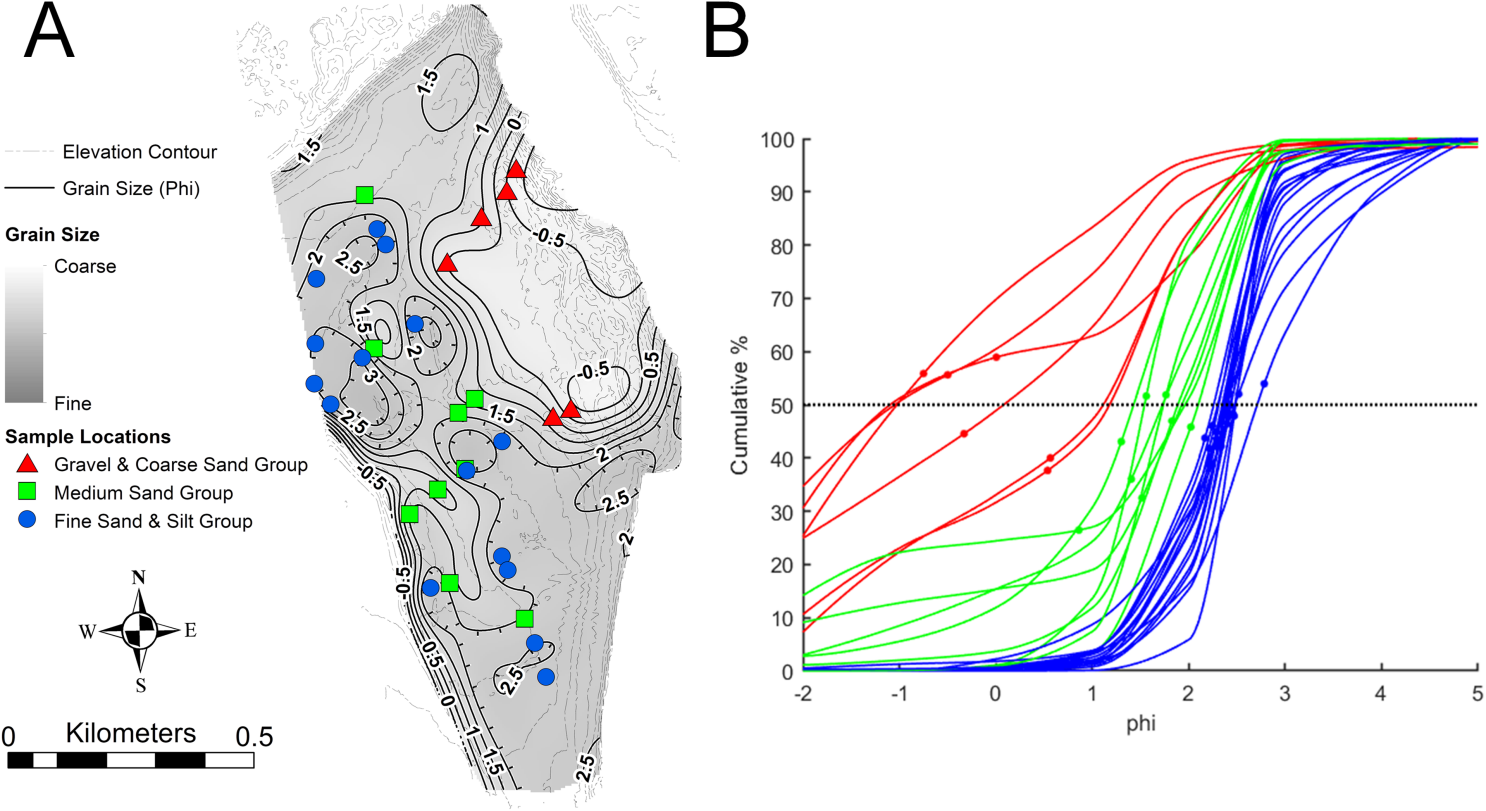
Sediment grain size on the modern tidal flat at Lubec, Maine. (A) Sediment mean grain size contours, in phi (ø) where ø = −log_2_(*D*/*D*_0_) and *D* is the intermediate-axis grain diameter of ellipsoidal-shaped grains, rendered dimensionally consistent by division by the reference diameter *D*_0_ (equal to one mm). Shading indicates fine to coarse gradation. Sample locations are color-coded for (B). Elevation contour interval 0.5 m. (B) Cumulative grain size distribution in phi units for samples keyed by color in (A). Mean values are marked by small dots. Grain size distributions with mean values above the 50% median line (dotted) are fine skewed, those below are coarse skewed.

**Figure 4:**
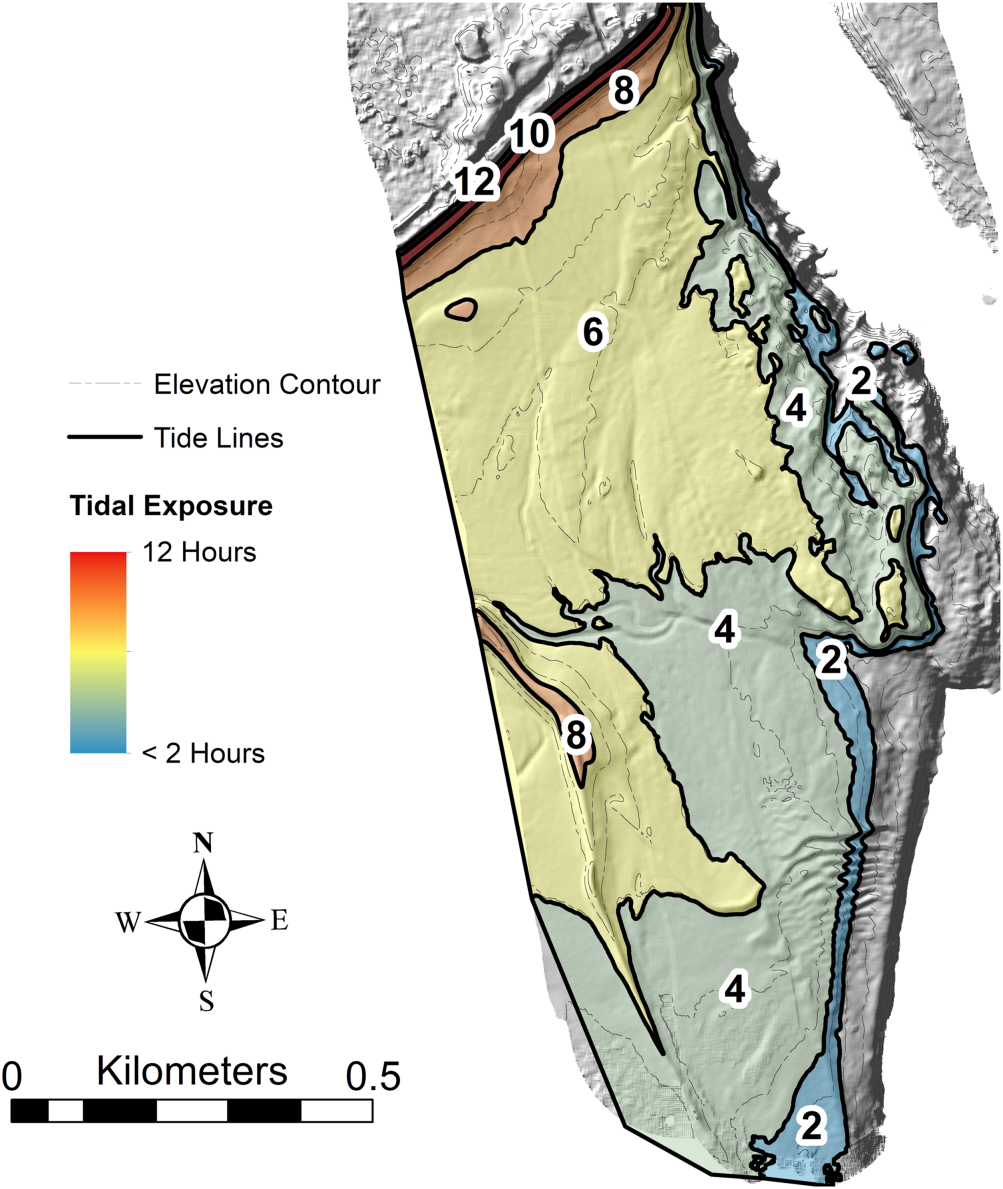
Hours of tidal exposure on the modern tidal flat at Lubec, Maine. Numbers represent maximum hours of exposure between contours per tidal cycle. Underlying image is a hillshade of the Lidar topobathymetry, with elevation contours (contour interval 0.5 m).

The type and abundance of organisms on the surface of the tidal flat during exposure are influenced by both the total time of exposure and the grain size of the substrate. Surface sediments are heterogeneous, ranging from fine gravel to silt. Within the study area (Fig. 3), fine gravel sediments predominate along a narrow ridge to the southwest (remnants of a Pleistocene esker) and on a mounded area to the northeast (which may be partly derived from dredge fill from the adjacent channel). The remaining area is characterized by medium to fine sand and silt. Some areas of the tidal flat experience up to 12 h of exposure each tidal cycle (high tide to high tide), although most of the area experiences between 4 and 6 h of exposure (Fig. 4). Exposure is the predominant factor defining the distribution of intertidal fauna (Dashtgard & Gingras, 2005), and thereby surface traces. Besides the physical effects of temperature variability, evaporation, and rainfall, exposure increases predation pressure. The apex natural predator on the tidal flat is the herring gull, *Larus argentatus smithsonianus*, followed by smaller shorebirds. The feeding gulls favor crabs (Ellis et al., 2005), including the species *Cancer borealis*, *Cancer irroratus*, and *Carcinus maenas*, and the smaller shorebirds prey on smaller crustaceans (Hamilton, Diamond & Wells, 2006).

**Figure 5:**
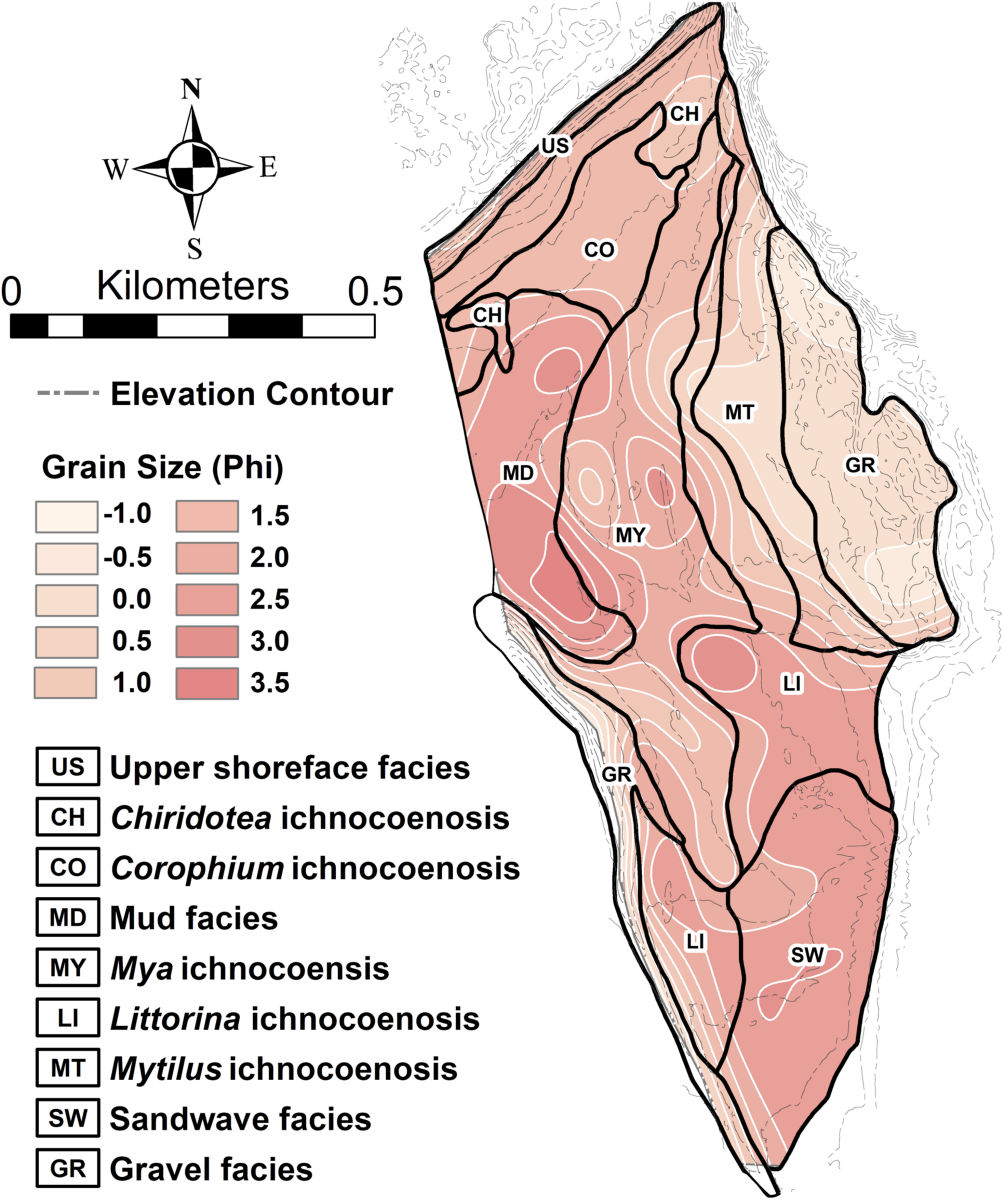
Ichnocoenoses and sedimentologic facies delineation on the modern tidal flat at Lubec, Maine. Close correspondence with sediment grain size is indicated by shading and light contours. Darker contours are elevation derived from Lidar coverage (contour interval 0.5 m).

The interaction of grain size and exposure time results in a distinct zonation of the flat (Fig. 5). The study area can be divided into five distinct ichnocoenoses characterized by surface traces (epichnia) and named for the single, most characteristic trace maker; and four sedimentologic facies defined by gross grain texture or hydrodynamic characteristics, but lacking significant surface traces. The latter are represented as facies rather than ichnocoenoses because epichnia are sparsely distributed and unlikely to be preserved.

### Upper shoreface facies

The substrate of the upper shoreface in the study area (more than 8 h exposure per tidal cycle) is primarily poorly sorted medium sand with abundant cobbles, some reaching boulder size. The mean grain size, excluding the cobbles, is 1.37 ø (0.39 mm), with a sorting index of 1.26 ø, and very coarse skew (skewness index −0.45). The surface is characterized by sea wrack left after every high tide. The wrack comprises mostly rockweed, *Ascophyllum nodosum*, but also includes a variety of kelp and other algal species. The wrack is ecologically important (Behbehani & Croker, 1982; Colombini & Chelazzi, 2003; MacMillan & Quijón, 2012; Stenton-Dozey, 1983) and several species of talitrid and hyalid amphipod crustaceans are abundant in and beneath it (Video S1), but leave no preservable traces. The predominant epichnial traces are those of vehicles and footprints of humans and dogs, that is, large terrestrial animals. The upper shoreface represents the eroding edge of moraine deposits, but attachment of cobbles to the algae and sediment entrapment in floating winter ice washed onto shore have been shown to be important depositional mechanisms in the Lubec embayment (Kelley, Belknap & Walsh, 2015). However, no drag marks from attached cobbles are observed on the upper shoreface because the seaweed is not moved by wind or current after settling on the beach.

### *Chiridotea* ichnocoenosis

The lower shoreface is delineated by a distinct break in slope, forming a gently sloping plane between the steep upper shoreface and the nearly horizontal tidal flats. The substrate is poorly sorted, medium sand, with mean grain size 1.43 ø (0.37 mm), sorting index 1.23 ø, and very coarse skew (skewness index −0.47). A variety of small crustaceans, notably the predatory (McDermott, 2005) isopod *Chiridotea coeca*, inhabit the sandy upper reach of the flat (6–8 h exposure per tidal cycle). This isopod is known to be selective for a medium grain size substrate (Griffith & Telford, 1985) and is abundant in this zone. The pelecypod mollusk *Macoma balthica* is also locally common in this zone, leaving a distinctive star-shaped feeding trace on the sediment surface (Brafield & Newell, 1961). Some small sand bars, topobathymetrically separated from the shoreline by minor sloughs, are also characterized by surface traces of *Chiridotea* and included in this ichnocoenosis.

**Figure 6:**
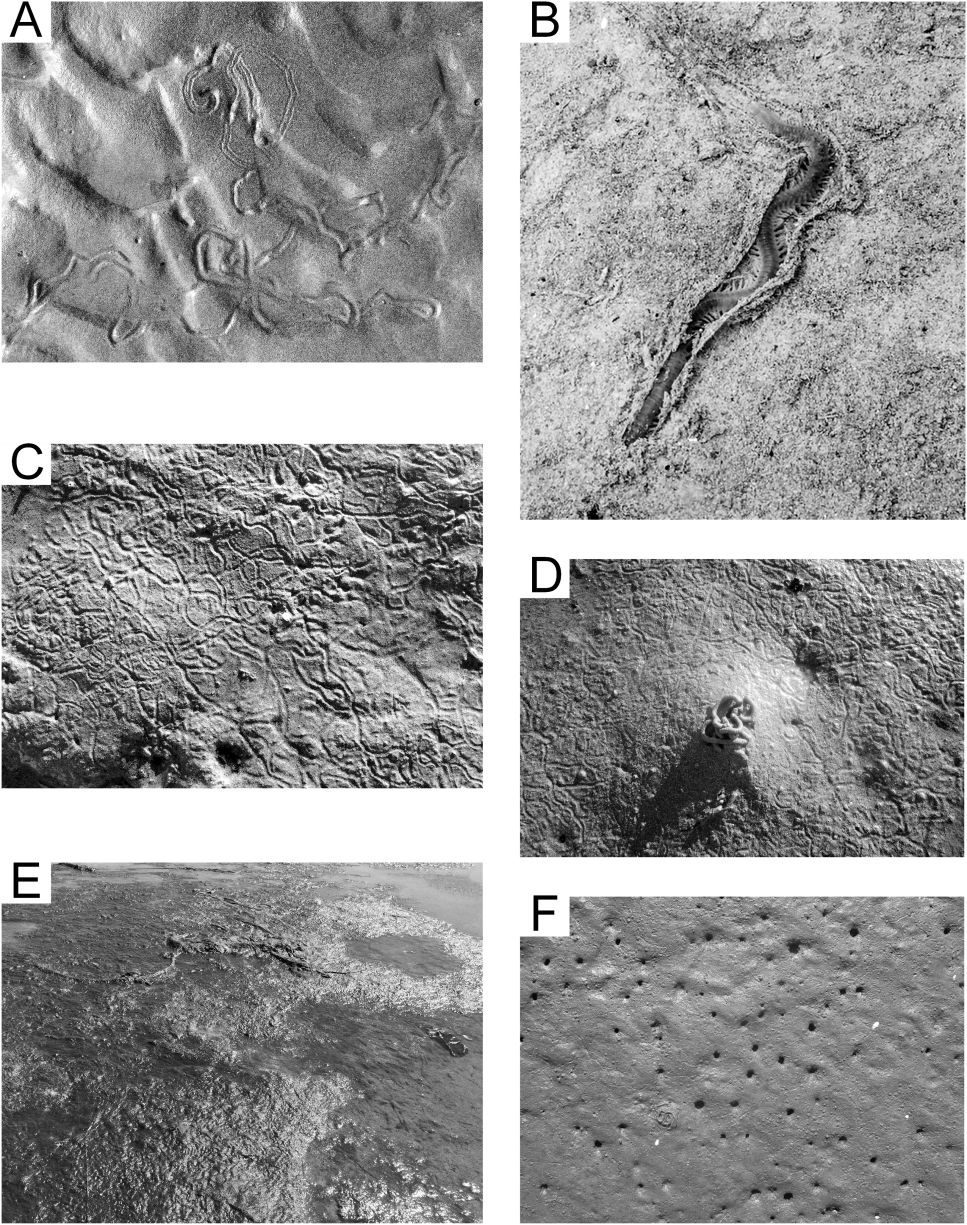
Epichnial traces on the modern tidal flat at Lubec, Maine. (A) *Chiridotea* ichnocoenosis. Trails of *Chiridotea coeca* on wet (image bottom) and dry (image top) sand. (B) *Chiridotea* ichnocoenosis. Active trail of *Nereis* sp. visible through the biofilm coating on a sand surface. (C) *Corophium* ichnocoenosis. Trails of *Corophium volutator* on wet sand. (D) *Corophium* ichnocoenosis. Fecal cast of *Arenicola marina* with trails of *Corophium volutator* on wet sand. (E) Mud facies. Filamentous green algae mat covering saturated silt and fine sand. (F) *Mya* ichnocoenosis. Siphon openings of *Mya arenaria* intersecting sand surface.

The surface trace produced by *Chiridotea coeca* initially consists of two parallel continuous marks with raised outside edges averaging four mm apart. These parallel marks deteriorate within minutes into a single groove with raised edges in damp fine-grained sand (Fig. 6A). This isopod essentially plows its way through the sediment, creating a small mounding of sediment at the forward end of the trail, which completely covers the animal (Video S2). Trails are generally meandering and densely looped to convolute, and concentrated in the troughs between ripples (Hauck, Dashtgard & Gingras, 2008). A thin, continuous biofilm is evident when the sand surface is wet, and tends to break up into small masses with entrained sediment that settle into the ripple troughs near the end of the exposure period. Nereid polychaetes are sometimes found moving through the biofilm when only a few millimeters of water cover the sediment (Fig. 6B; Video S3).

### *Corophium* ichnocoenosis

The amphipod *Corophium volutator* is abundant in poorly sorted medium sand in the upper reach of the flat (5–6 h exposure per tidal cycle). Mean grain size is 1.37 ø (0.39 mm) with a sorting index of 1.25 ø, and fine skew (skewness index 0.03). In summer months, the amphipods leave their burrows to breed (Forbes et al., 1996), and surface trails cover significant areas of the flats within this ichnocoenosis (Fig. 6C). The trails form shallow continuous grooves averaging 2.5 mm in width, with slightly raised edges. The substrate is too coarse for development of the beaded pattern described by Häntzschel (1939), which is more representative of European populations where the species inhabits finer substrate (Meadows, 1964). Small (1.0 mm) fecal pellets of uncertain origin often cover the sediment surface as well. A distinctive system of lined, U-shaped burrows (Video S4), penetrating only a few centimeters within the sediment, is developed by *Corophium volutator* (Meadows & Reid, 1966). The polychaete annelid *Nephtys caeca* is observed in sediment with *Corophium volutator*, and forms horizontal burrows that intersect with the *Corophium volutator* burrows. These and other nereids are known to prey on *Corophium* species (Jensen & André, 1993). The polychaete *Arenicola marina*, which is absent from the *Chiridotea* ichnocoenosis, is common, but with variable density. *Arenicola marina* creates U-shaped burrows penetrating as deep as 15 cm into the sediment (Christian et al., 2010) with distinctive mounds (Fig. 6D) at the excretory openings of the burrows (Plante, 2010).

### Mud facies

The mud facies has few, if any, surface traces because the sediment surface is covered with algal mats (Fig. 6E), comprising primarily filamentous green algae (*Rhizoclonium* spp., *Acrosiphonia* spp., etc.) and brown algae (*Fucus* spp.). The visible organic content is high and the sediment is a moderately well sorted fine sand to silt, with mean grain size 2.39 ø (0.19 mm), sorting index 0.71 ø, and coarse skew (skewness index −0.11). The sediment maintains a fully-saturated state even during low tides (4–6 h exposure per tidal cycle), represents the “soupy mud” environment described by Dashtgard, Gingras & Pemberton (2008), and is not a significant substrate for epichnial traces.

### *Mya* ichnocoenosis

The middle or mid-tidal reach sediment (4–5 h exposure per tidal cycle) is moderately sorted medium sand with mean grain size 1.73 ø (0.30 mm), sorting index 0.90 ø, and minimal skew (skewness index −0.01). The epichnia comprise primarily burrow openings of pelecypod mollusks (notably *Mya arenaria*, but *Macoma balthica* is locally common), with uniformly scattered mounded burrow openings of the annelid *Arenicola marina* (Fig. 6F) and their associated fecal castings. The infauna also includes nereid polychaetes and is equivalent to the “*Mya-Nereis virens* Biome” of Newcombe (1935b). Sedimentologic study in this zone is hampered by the extensive reworking caused by individuals clam digging (restricted by permit) for this economically valuable mollusk.

### *Littorina* ichnocoenosis

**Figure 7:**
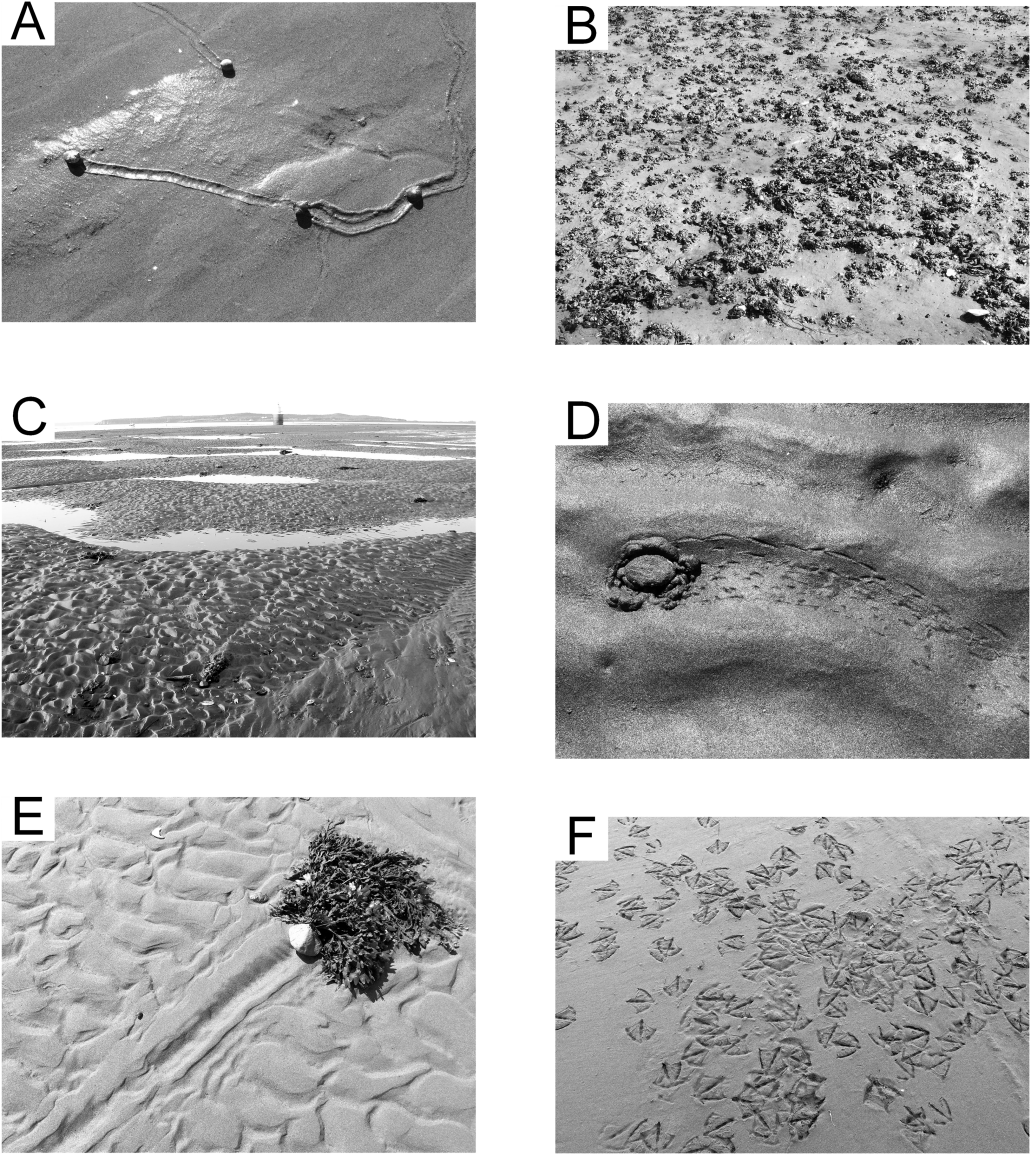
Epichnial traces on the modern tidal flat at Lubec, Maine. (A) *Littorina* ichnocoenosis. Active trails of *Littorina littorea* on wet sand. (B) *Mytilus* ichnocoenosis. Aggregations of *Mytilus edulis* on muddy sediment. (C) Sandwave facies. Mega-ripples covering surface of large wavelength sandwaves with standing water in interdune troughs. (D) Sandwave facies. Trail from crab *Carcinus maenas* stranded by a falling tide. (E) Sandwave facies. Drag mark from a cobble with attached seaweed. (F) Congregating tracks of *Larus argentatus smithsonianus* characteristic of “feeding frenzy” scavenging behavior.

The lower portion of the middle reach (2–5 h exposure per tidal cycle) is moderately sorted medium sand, with mean grain size 1.72 ø (0.30 mm), sorting index 0.81 ø, and minimal skew (skewness index −0.03). The surface is generally unmarked except for the trails of the invasive gastropod mollusk *Littorina littorea* (Fig. 7A). These distinctive, grooved trails average one cm in width and are generally straight, often extending several meters from start to end. The mollusks remain shallowly buried in the sediment until exposed by a falling tide. These trails may be singular or tightly grouped depending on the local density of the mollusks, and mating activity often brings trails together (Johannesson et al., 2010).

### Gravel facies

The gravel facies is characterized by poorly sorted fine gravel to coarse sand substrate with mean grain size −1.65 ø (3.1 mm), sorting index of 2.04 ø, and coarse skew (skewness index −0.12). Two separate parts of the study area are represented by this facies. A gravel bar on the southwest boundary of the study area, the remnant of a glacial esker, is very coarse and compacted, experiences from 4 to 8 h of exposure per tidal cycle, and is essentially devoid of any surface traces or infauna. The other area is topobathymetrically low (0–4 h exposure per tidal cycle), but hummocky and irregular. This area may in part represent material dredged from the Lubec channel, but the gravel is in places less than one m in thickness and underlain by dense clay of the Late Pleistocene Presumpscot Formation (Oldale, 1989). The gravel surface prevents the generation of any surface traces, although there is an abundant infauna (primarily polychaete annelids). The irregular surface with permanent tidal pools supports a depauperate fauna of subtidal organisms (including decapod crustaceans, echinoderms, and motile mollusks).

### *Mytilus* ichnocoenosis

A band of sediment surrounding the gravel ichnocoenosis (2–4 h exposure per tidal cycle) represents a mix of poorly sorted coarse sand and scattered medium gravel. The mean grain size is 0.29 ø (0.82 mm), with sorting index of 1.64 ø, and minimal skew (skewness index −0.03). The gravel is an attractive attachment substrate for the pelecypod mollusk *Mytilus edulis*, and the surface is densely covered with small agglomerations of them (Fig. 7B). During spring and early summer, the zone is host to abundant green and brown algae, which are reduced in extent and prevalence later in the season. The gastropod *Littorina littorea* is common when algae are present, representing the opportunistic nature of this species (Wilhelmsen & Reise, 1994). The polychaete *Arenicola marina* is present in low density and leaves the only significant surface trace with burrow mounds and tubular fecal casts. This zone, in combination with the *Littorina* ichnocoenosis, is equivalent to the “*Balanus-Mytilus edulis* Association” of Newcombe (1935a) or “*Balanus-Littorina-Fucus* Biome” of Newcombe (1935b), although the barnacle *Balanus* is only a minor component in the study area because of the relative scarcity of large rocks for attachment.

### Sandwave facies

Intertidal sandwaves are a characteristic feature of the outer littoral zone of the Bay of Fundy region (Dalrymple, 1984). In the study area, sandwaves average 0.4 m in height and vary from 12 to 18 m crest to crest. This zone is submerged longer than any other part of the flat and experiences strong tidal currents with shifting mega-ripples up to 10 cm in height capping the sandwaves (Fig. 7C). Dominant current direction is toward the southwest (aligned with the outgoing tide), resulting in large-scale crossbeds on a scale equivalent to that of the height and wavelength of the sandwaves, modified by small-scale crossbeds developed from the mega-ripples. The sediment is well sorted fine sand with mean grain size 2.20 ø (0.2 mm), sorting index of 0.44 ø, and coarse skew (skewness index −0.16), and usually remains nearly or fully saturated. Decapod crustaceans, gastropods, and echinoderms are found in the lower reach of the flat (0–4 h exposure) where they are often stranded by spring tides. Infaunal annelids, both polychaetes and oligochaetes, are abundant in this zone, and, locally, tubes of the annelid *Clymenella torquata* form dense accumulations—a pattern seen throughout the Bay of Fundy region (Featherstone & Risk, 1977). Surface traces of invertebrates are sparsely scattered, comprising trails of *Littorina littorea* and other gastropods, decapod crustaceans including *Carcinus maenas* (Fig. 7D) and *Pagurus* spp., and common drag marks from cobbles bound to drifting seaweed (Fig. 7E). Spring low tides expose the sand dollar echinoid *Echinarachnius parma* for short periods, and these often leave short, wide trails. The most prevalent surface traces are footprints of the gull *Larus argentatus smithsonianus* (Fig. 7F), the apex predator/scavenger on the tidal flats.

### Paleoichnocoenoses

**Figure 8:**
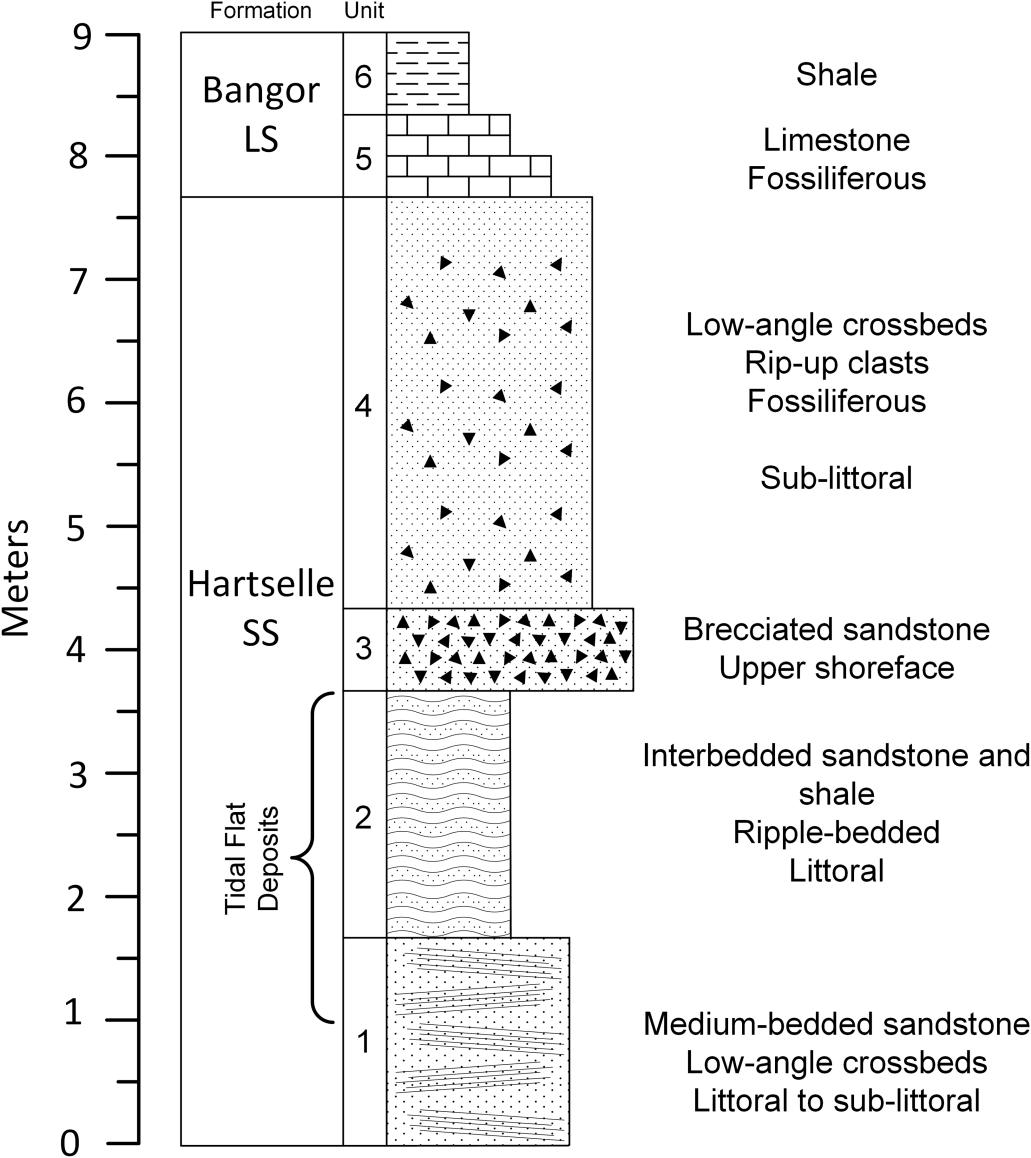
Geologic column of the upper portion of Hartselle Sandstone exposure at Fielder Ridge, Colbert County, Alabama. Datum is arbitrary and does not mark the base of the Hartselle Sandstone (which is no longer exposed at this site).

**Figure 9:**
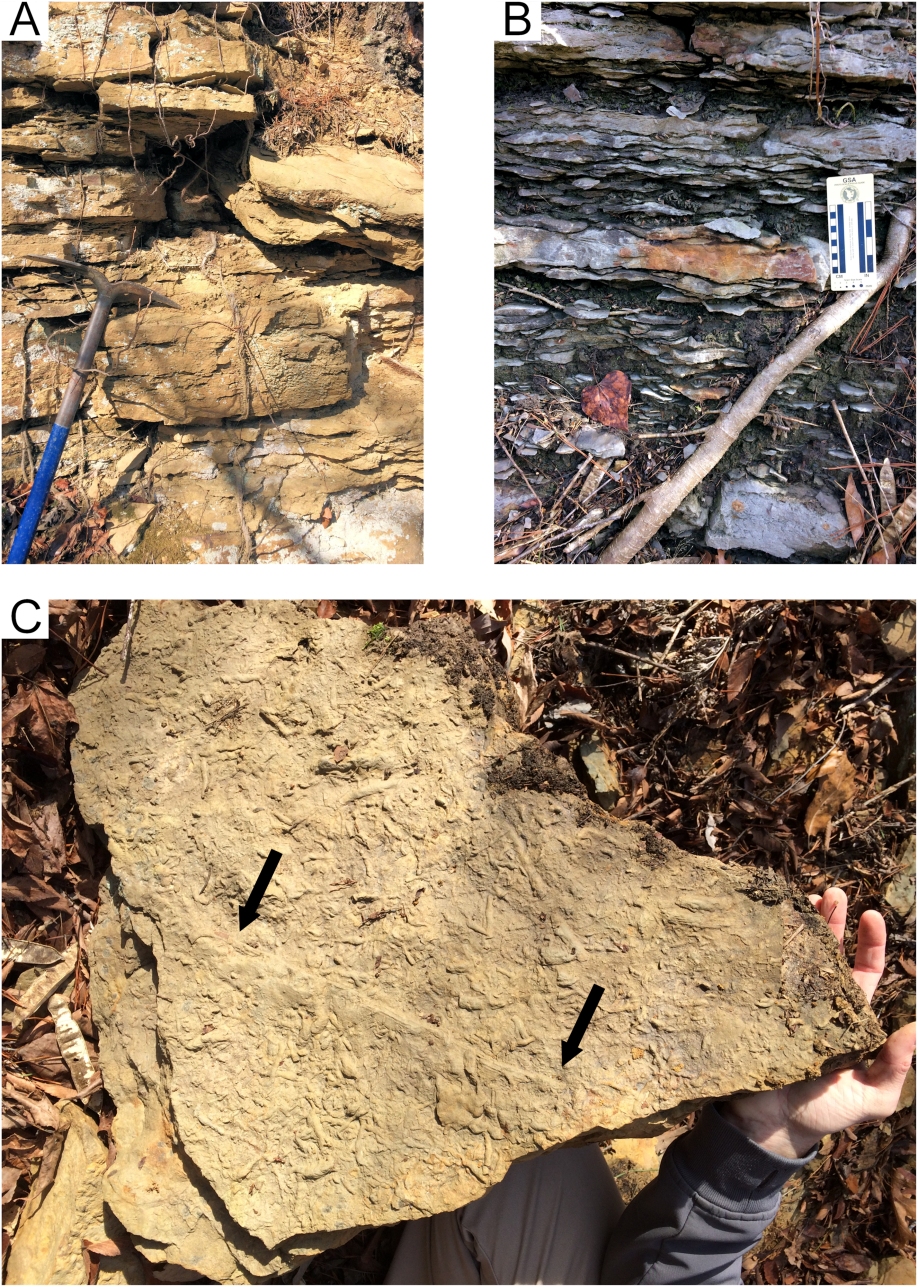
Hartselle Sandstone tidal flat deposits, Fielder Ridge, Colbert County, Alabama. (A) Unit 1, medium-bedded quartz sandstone. Head of pick approximately 35 cm tip to tip. (B) Unit 2, interbedded fine-grained quartz sandstone and interbedded shale. Note the characteristic lenticular, starved ripple bedding of this unit. (C) Slab from Unit 1, hypichnial view, showing abundant traces and long drag mark (arrows). Hand for scale.

The geologic column covering the upper portion of the Fielder Ridge section of the Hartselle Sandstone exposure (Fig. 8) comprises a regressive succession of subtidal, intertidal, and upper shoreface deposits, followed by a rise in relative sea level and transition into the Bangor Limestone. The base of the Hartselle is no longer exposed here (because of slumping and vegetation overgrowth), but a complete section was described by Kopaska-Merkel & Rindsberg (2016). Within the study section, the lower unit (Unit 1) is a medium-bedded (10–35 cm bed thickness), fine- to medium-grained quartz sandstone (Fig. 9A), with low-angle directionally-oriented and flaser crossbeds, and abundant hypichnial traces and drag mark casts. It is overlain by approximately two m of fine-grained sandstone (one to three cm bed thickness) with shale interbeds (Unit 2). The sandstone has a lenticular to wavy ripple-bedding (Fig. 9B), with flaser bedding expressed in the thicker beds. This unit contains abundant epichnial and hypichnial trace fossils, with occasional preservation of sub-vertical to vertical burrows and internal crossbedding. The ripple-bedded unit is overlain by a coarse-grained quartz sandstone containing abundant brecciated clasts of sideritic claystone and calcareous fossil debris (Unit 3). This unit grades upward into similar coarse-grained sandstone with low-angle cross-stratification (Unit 4). These coarse units lack any unequivocal trace fossils. The base of the Bangor Limestone is a micritic limestone with occasional hypichnial traces (Unit 5), grading upward into calcareous shale (Unit 6).

#### Unit 1: Planolites-Phycodes paleoichnocoenosis

**Figure 10:**
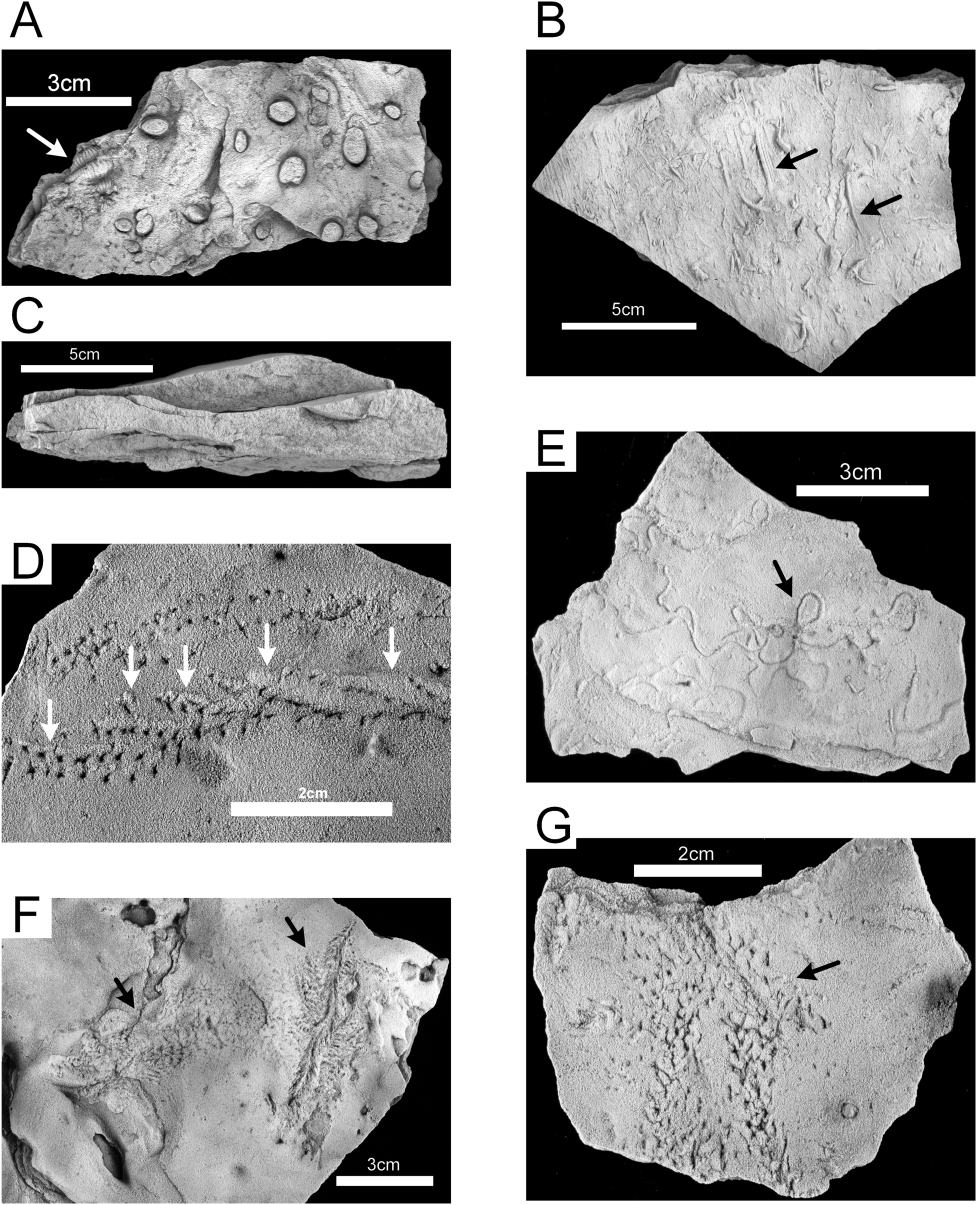
Hartselle Sandstone trace fossils. (A) *Lockeia cordata*, with trilobite body fossil (arrow), epichnial, MMNS IP-10016. (B) Drag mark casts (arrows), hypichnial view, MMNS IP-10014. (C) Cross-section of rippled sandstone, MMNS IP-10013. (D) *Diplichnites gouldi*, showing displaced tracks and push-up mounds (arrows) as animal moved upslope on loose sand, epichnial view, MMNS IP-10025. (E) *Haplotichnus ornatus* (arrow), epichnial view, MMNS IP-10021. (F) *Nereites*? isp. (arrows), epichnial view, MMNS IP-10020. (G) *Petalichnus*? isp. (arrow), epichnial view, MMNS IP-10024. All specimens whitened with ammonium chloride.

The lower, medium-bedded quartz sandstone unit (Unit 1) is characterized by abundant hypichnial traces, attributed by Rindsberg (1994) to *Planolites*, *Phycodes*, *Ptychoplasma*, and *Dactylophycus*, and drag mark casts (Fig. 9C). Epichnial traces are uncommon and poorly preserved. The relatively thick bedding allows large slabs to be removed and examined, but the beds thin upward and grade into the ripple-bedded unit. The thinner beds often have abundant pelecypod mollusk burrows (*Lockeia cordata)*. Trilobite pygidia and small brachiopods may co-occur with the burrows (Fig. 10A). Drag mark casts are also common in these thinner beds (Fig. 10B).

#### Unit 2

The ripple-bedded sandstone and shale unit (Unit 2) is characterized by abundant trace fossils, both epichnial and hypichnial, with some preservation of endichnial burrows. Rarely are traces reworked by younger traces. The beds in Unit 2 represent, for the most part, starved ripples, with limited influx of coarser clastic material. The interbedded shales in Unit 2 contain pyrite, and some portions of the sandstone contain oxidized pyrite concretions. Fine carbonized plant remains are present in some ripple troughs. The thin, lenticular to wavy beds (Fig. 10C) are enveloped in soft shale and can be readily extracted from the outcrop, which has aided in the preservation of the traces. Three distinct paleoichnocoenoses are delineated within this unit. There is no observed pattern to the vertical sequence of individual zones.

### *Diplichnites* paleoichnocoenosis

**Figure 11:**
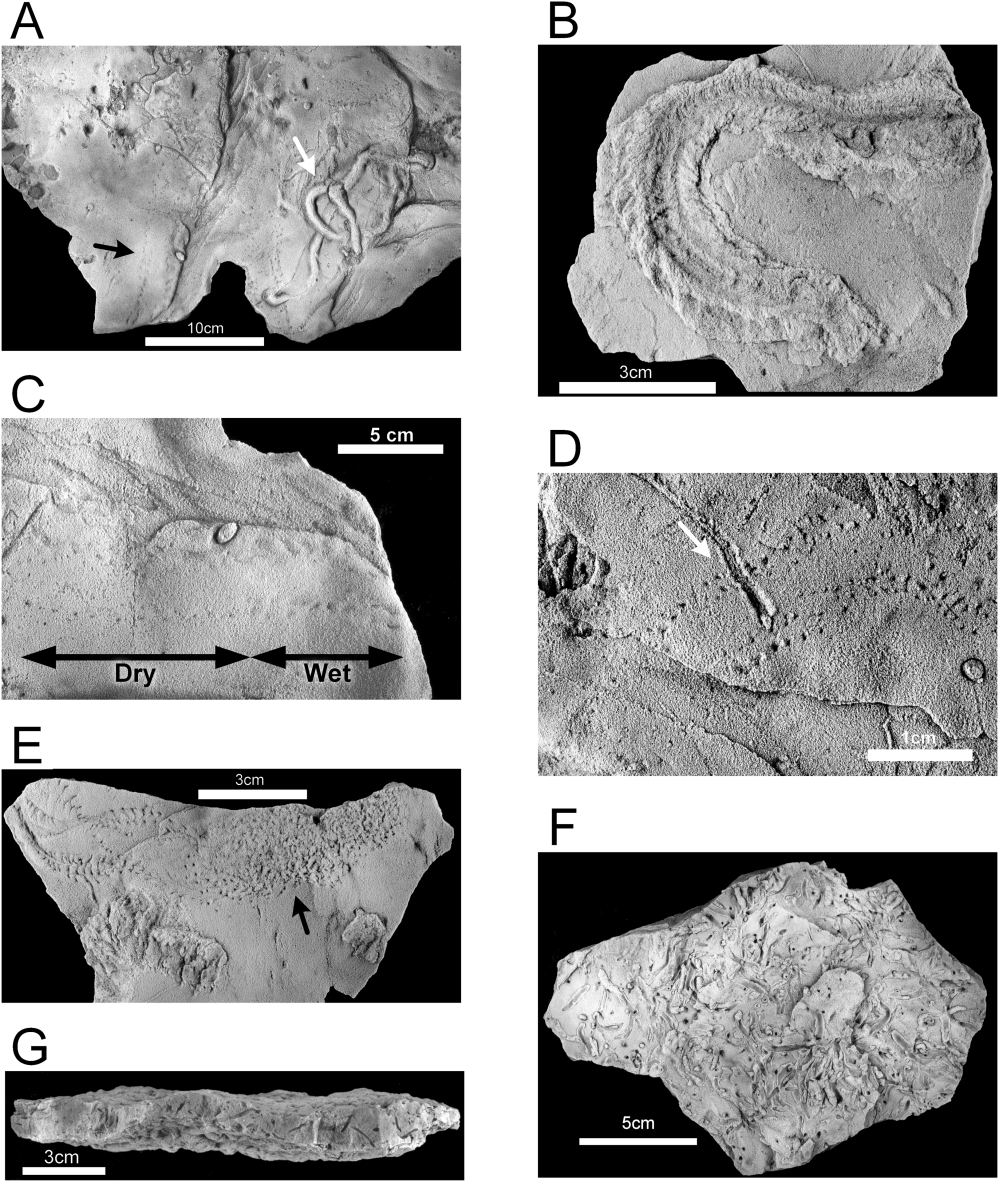
Hartselle Sandstone trace fossils. (A) Fecal cast (white arrow), with *Diplichnites gouldi* (black arrow), epichnial view, MMNS IP-10019. (B) *Nereites missouriensis*, showing possible biofilm, epichnial view, MMNS IP-10022. (C) *Diplichnites gouldi*, showing possible wet to dry tracks (range marked by arrows), epichnial view, MMNS IP-10019. (D) *Diplichnites gouldi* with crossing trace (aligned with arrow), epichnial view, MMNS IP-10018. (E) *Diplichnites gouldi*, chaotic tracks (arrow) indicative of “feeding frenzy” behavior, epichnial view, MMNS IP-10023. (F) *Hartsellea sursumramosa*, epichnial view, MMNS IP-10012. (G) *Hartsellea sursumramosa*, lateral view showing burrows, epichnial surface is up, MMNS IP-10015. All specimens whitened with ammonium chloride.

The epichnial traces of *Diplichnites gouldi* (Fig. 10D) are ubiquitous in this paleoichnocoenosis. This trace was described by Rindsberg (1994) as *Petalichnus* isp., but the distinctive double series of parallel rows of blunt tracks lacking any midline trace are definitive for *D. gouldi* (Smith et al., 2003), although there is disagreement regarding the ichnotaxonomy (Minter & Braddy, 2009). Other traces include a looping, self-crossing trail described as *Haplotichnus ornatus* by Rindsberg & Kopaska-Merkel (2005) (Fig. 10E), trails attributed to *Uchirites* by Rindsberg (1994), a variety of unidentified tracks (Figs. 10F and 10G), and tubular fecal castings (Fig. 11A). This paleoichnocoenosis is observed in the thin (one to three cm), lenticular-bedded sandstone with shale interbeds. It corresponds, in part, with the *Hartsellea* assemblage described by Rindsberg (1994) by assignment of the actual *Hartsellea*-bearing beds into a separate paleoichnocoenosis.

### *Hartsellea* paleoichnocoenosis

The *Hartsellea* paleoichnocoenosis as defined here is more restrictive than the *Hartsellea* assemblage described by Rindsberg (1994) and is characterized by abundant, nearly monospecific collections of burrows and trails of the ichnospecies *Hartsellea sursumramosa*. The epichnial traces are lined (Fig. 11F). The burrows of *Hartsellea sursumramosa* are not strictly vertical (Fig. 11G). Hypichnial traces cannot rule out connection to multiple openings. The burrows do not penetrate more than one or two cm into the sandstone. This paleoichnocoenosis is associated with thicker (two to five cm), wavy-bedded sandstone.

### *Lockeia* paleoichnocoenosis

**Figure 12:**
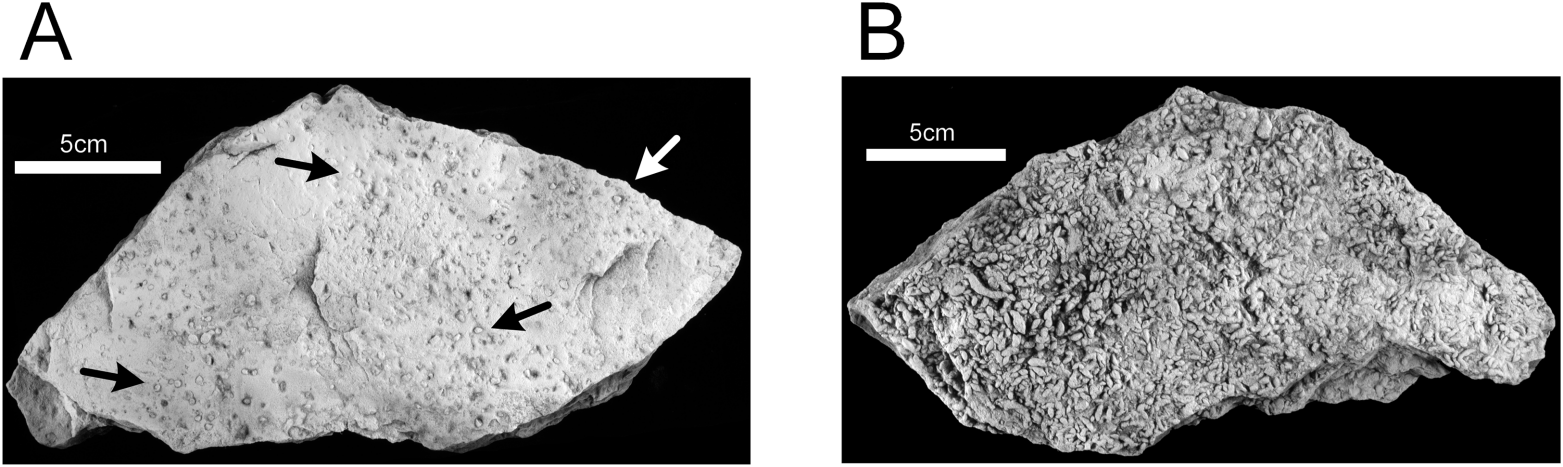
Hartselle Sandstone trace fossils. *Lockeia siliquaria*, MMNS IP-10017. (A) Epichnial view, with ovate openings of siphonal tubes (arrows). (B) Hypichnial view. Specimens whitened with ammonium chloride.

The *Lockeia* paleoichnocoenosis corresponds to the *Lockeia* assemblage described by Rindsberg (1994) and is characterized by abundant, nearly monospecific collections of burrows of *Lockeia siliquaria* (Figs. 12A and 12B). Epichnia comprise ovate siphonal openings. These traces are significantly smaller than the ichnospecies *Lockeia cordata* found in the lower sandstone Unit 1 and penetrate one or two cm into the sandstone. This paleoichnocoenosis is associated with thicker (two to five cm), horizontally-bedded sandstone.

#### Unit 3

The coarse-grained quartz sandstone containing abundant brecciated clasts of sideritic claystone and calcareous fossil debris (Unit 3) is characterized by trough and tabular crossbed sets two to three cm thick, large clasts up to four cm in diameter, with disarticulated crinoid and blastoid columnals, broken fenestrellid bryozoans and small, fragmented brachiopods. No ichnofossils were observed.

#### Unit 4

The uppermost Hartselle Sandstone beds (Unit 4) comprise coarse-grained quartz sandstone with enough echinoderm debris to be termed calcareous. The thin (one to two cm) beds are collected into bedsets up to one m thick that have a low-angle crossbedded fabric in outcrop. Claystone clasts are a minor component in some beds. No ichnofossils were observed.

## Discussion

The depositional environments of the Hartselle Sandstone at Fielder Ridge can be interpreted by comparison with the characteristics of a modern macro-tidal flat observed at the Lubec study area.

### Wind and wave influence

In general, wave dominance results in coarser sediment, including reworking of lithified material, while tide dominance is associated with silt and clay deposition. The distinction of degree between wave vs. tidal dominance of a depositional environment is difficult to determine at a small scale, but at a large scale, tidal dominance is characterized by a series of offshore banks and ridges (Anthony & Orford, 2002), a depositional pattern represented regionally by that of the Hartselle Sandstone (Fig. 1B). Wave vs. tide dominance can also be evaluated in the vertical sequence of strata (Fig. 8). The bidirectionally-oriented and flaser crossbeds in the lower Unit 1, and the rhythmically bedded lenticular and wavy ripples of Unit 2, support the interpretation of tidal current rather than wave influence. The coarse, brecciated Unit 3 of the outcrop, equivalent to the multiple crossbed set facies of Thomas & Mack (1982), has the characteristics of wave dominance and is interpreted as an upper shoreface environment exposed to wind and wave action. The overlying Unit 4, containing an increased amount of disarticulated fossils and laminated beds collected into bedsets with low-angle crossbedded fabric, is interpreted as a sublittoral facies resulting from the progradation and reworking of shoreface deposits associated with a rise in sea level. It is equivalent to the horizontally laminated sandstone facies of Thomas & Mack (1982).

### Brackish-water influence

The high diversity and good preservation of ichnofossils in the Hartselle Sandstone at Fielder Ridge are evidence for the lack of brackish-water influence. In modern estuarine settings, biologic traces from the fluvial-tidal transition zone have little preservation potential (Shchepetkina et al., 2016). In fact, zones of fresh and salt water mixing are characterized by a general impoverishment of ichnodiversity (Gingras, MacEachern & Dashtgard, 2012). Sedimentologically, zones of fluvial-tidal mixing are muddy and clay-rich because of flocculation of transported clays in the presence of saltwater. Although Unit 2 of the Hartselle includes bed sets of sand-starved ripples, the unit is overall predominantly quartz sandstone with only thin beds of clay shale. The starved ripples indicate no significant sandy sediment influx from a nearby fluvial or estuarine source. The lower Unit 1 comprises quartz sandstone and lacks significant clay or shale.

All four of the delineated units of the Hartselle Sandstone preserve some degree of crossbedding, but lack the gross fabric described as inclined heterolithic stratification or IHS (Thomas et al., 1987) that characterizes estuarine tidal deposits (Gingras, MacEachern & Dashtgard, 2012). The Hartselle beds are atypical for a soft-substrate *Skolithos* ichnofacies, lacking the extensive vertical burrowing that is considered diagnostic for brackish-water conditions (Buatois et al., 2005; Hauck et al., 2009).

### Distribution of distinct microenvironments

The Lubec study covered an area of approximately 60 ha. This is a small section of coastal zone, yet it can be subdivided into nine distinct microenvironments, each represented by characteristic fauna and flora or sedimentologic facies. The diversity of traces is greatest proximal to the shoreface, where sediment stability is maximized, emphasizing the importance of physical energy as an additional factor in a macro-tidal regime. The delineation of four distinct ichnocoenoses in Units 1 and 2 plus the lithologically defined Units 3 and 4 within the Hartselle at Fielder Ridge are comparable to the diversity of environments found in the modern study area (Table 1).

**Table 1:** Comparison of modern ichnocoenoses and sedimentologic facies at Lubec, Maine with paleoichnocoenoses and lithologic units in the Hartselle Sandstone at Fielder Ridge, Alabama.

Hartselle Sandstone		Lubec embayment
Fielder Ridge unit	Paleoichnocoenosis or facies	Modern ichnocoenosis or facies
(No equivalent)		*Mytilus* ichnocoenosis
4	Littoral to sublittoral coarse sandstone and gravel, with marine fossils	Gravel facies
3	Coarse sandstone with abundant claystone clasts and broken fossil debris	Upper shoreface facies
2	*Diplichnites* paleoichnocoenosis	C*hiridotea* ichnocoenosis *Littorina* ichnocoenosis
	*Hartsellea* paleoichnocoenosis	*Corophium* ichnocoenosis
	Interbedded shales	Mud facies
	*Lockeia* paleoichnocoenosis	*Mya* ichnocoenosis
1	*Planolites-Phycodes* paleoichnocoenosis	Sandwave facies

In the lowest beds, the predominance of hypichnial, horizontal traces, their overall abundance and diversity, and inclusion of body fossils of marine organisms are representative of the *Cruziana* ichnofacies (Buatois & Mángano, 2011) and a sublittoral depositional environment. It is equivalent to the interbedded crossbedded and rippled sandstone facies of the Hartselle described by Thomas & Mack (1982). The upper part of this unit, at least, was likely situated within wave base, and the *Lockeia*-bearing beds probably represent the distal portion of tidal flats, analogous to the *Mya* ichnocoenosis of the modern Lubec flats. The lower portion, with low-angle crossbeds and preserved drag marks, indicate an environment similar to the sandwave facies at Lubec, which extends into the sublittoral zone.

The ripple-bedded Unit 2 preserves the best evidence for a meso- or macro-tidal marine environment. The ripple-bedded sandstone and shale unit (Unit 2) is equivalent to the rippled sandstone-mudstone facies of the Hartselle described by Thomas & Mack (1982) and the starved ripples indicate a relatively muddy nearshore environment with limited influx of coarser clastic material. The interbedded shales in Unit 2 contain pyrite, and some portions of the sandstone contain oxidized pyrite concretions, which is consistent with a shallow anoxic reduced zone similar to that present in the Lubec tidal flat. The shale beds are analogous to the modern mud facies at Lubec. The diversity of epichnial traces on the rippled sandstones conforms to similar traces found in the modern *Chiridotea*, *Corophium*, and *Mya* ichnocoenoses. The *Diplichnites* paleoichnocoenosis shares many characteristics with the modern *Chiridotea* ichnocoenosis. Modern trails of *Chiridotea coeca* closely resemble those of *Haplotichnus ornatus*, described by Rindsberg & Kopaska-Merkel (2005), and show some similarity with *Uchirites implexus.* The ichnogenus *Haplotichnus* was considered a junior synonym of *Treptichnus* by Getty & Bush (2017), who noted that *Haplotichnus ornatus* does not fit within *Treptichnus* nor the related ichnogenus *Gordia*. Regardless of nomenclature, the traces fall into the architectural design defined by Buatois et al. (2017) as simple horizontal trails, are analogous to similar traces characterizing the modern *Chiridotea* ichnocoenosis, and imply a proximal (near the shore) location on a tidal flat. Some traces, such as that of *Nereites missouriensis* (Fig. 11B), suggest the presence of an extensive biofilm as well (compare with Fig. 6B). The *Hartsellea* paleoichnocoenosis resembles the modern *Corophium* ichnocoenosis, with its dense collection of surface trails created by the amphipods leaving their burrows and seeking either food or mates. Rindsberg (1994) attributed the traces to a polychaete, analogous to *Neanthes virens* (= *Alitta virens*), also known from Bay of Fundy tidal flats. We interpret the traces as attributable to an amphipod-like arthropod analogous to *Corophium*. The epichnial traces are lined (Fig. 11F), but this could be a result of horizontal movement through biofilm. The burrows of *Hartsellea sursumramosa* are not U-shaped like those of *Corophium*, but are also not strictly vertical (Fig. 11G) and hypichnial traces cannot rule out connection to multiple openings. Whether or not the actual trace maker is an amphipod, this ichnocoenosis is thought to be analogous with the modern *Corophium* ichnocoenosis. The *Lockeia* paleoichnocoenosis is characterized by its presumptive molluscan trace maker. These traces are significantly smaller than the ichnospecies *Lockeia cordata* found in the lower sandstone Unit 1, and attributable to a smaller pelecypod mollusk burrowing only one or two cm into the sediment. Rindsberg (1994) suggested that the small size and dense accumulations represented a mortality event of a nursery population, but similarly dense accumulations of mature *Macoma balthica* and *Mya arenaria* occur today on the Lubec tidal flats. This ichnocoenosis is considered analogous with the modern *Mya* ichnocoenosis, and indicates a more distal location on the flat.

### Diversity of epichnia

A total of 23 separate ichnotaxa, most with at least some epichnial expression, were reported by Rindsberg (1994) from the Fielder Ridge locality. Collections from the exposure at Fielder Ridge have added additional undescribed ichnotaxa, with an estimated total of 25 or more distinct epichnia (as well as additional hypichnia). This number is comparable to the general variety of traces found at the Lubec study area and represents a similar degree of ichnodiversity.

### Degree of reworking

The ichnofossils preserved in Units 1 and 2 show almost no evidence of reworking by bioturbation, a trait common to most Hartselle trace fossils regardless of locality (Rindsberg, 1994). The presence of pyrite and limonite (oxidized pyrite) is evidence for shallow anoxic conditions in the original sediment. In the modern study area environment, only deep-burrowing mollusks (*Mya*) and annelids (*Arenicola*) with well-irrigated, relatively permanent vertical burrows open to the sediment surface inhabit this anoxic layer, thereby limiting the depth and character of any bioturbation. This is a pattern similar to that described by Baucon & Felletti (2013), where burrowing is restricted by the adaptation of organisms to the dysoxic environment. The extensive preservation of crossbedding in nearly all of the strata of Units 1 and 2 argues further against any significant bioturbation of the sediment. The presence of abundant epichnia in Unit 2 coupled with their poor preservation in Unit 1 supports reworking of the sediment surface by tidal currents in the more distal and marginally subtidal portion of the flat.

### Predation

One of the most distinctive and prevalent of the traces found in the ripple-bedded Unit 2 of the Hartselle is *D. gouldi*. This trace has been attributed to a myriapod (Smith et al., 2003), but there are several other interpretations in the literature (Getty et al., 2017). However, there are reasons to support the interpretation of the *D. gouldi* ichnofossils from the Hartselle Sandstone as myriapod traces. Myriapods are not strictly terrestrial and there are many living genera of littoral myriapods (Barber, 2009), in particular, littoral chilopods or centipedes. There are more than 40 modern species of geophilomorph centipedes known to tolerate immersion in salt water and that inhabit littoral environments (Barber, 2011, 2019).

**Figure 13:**
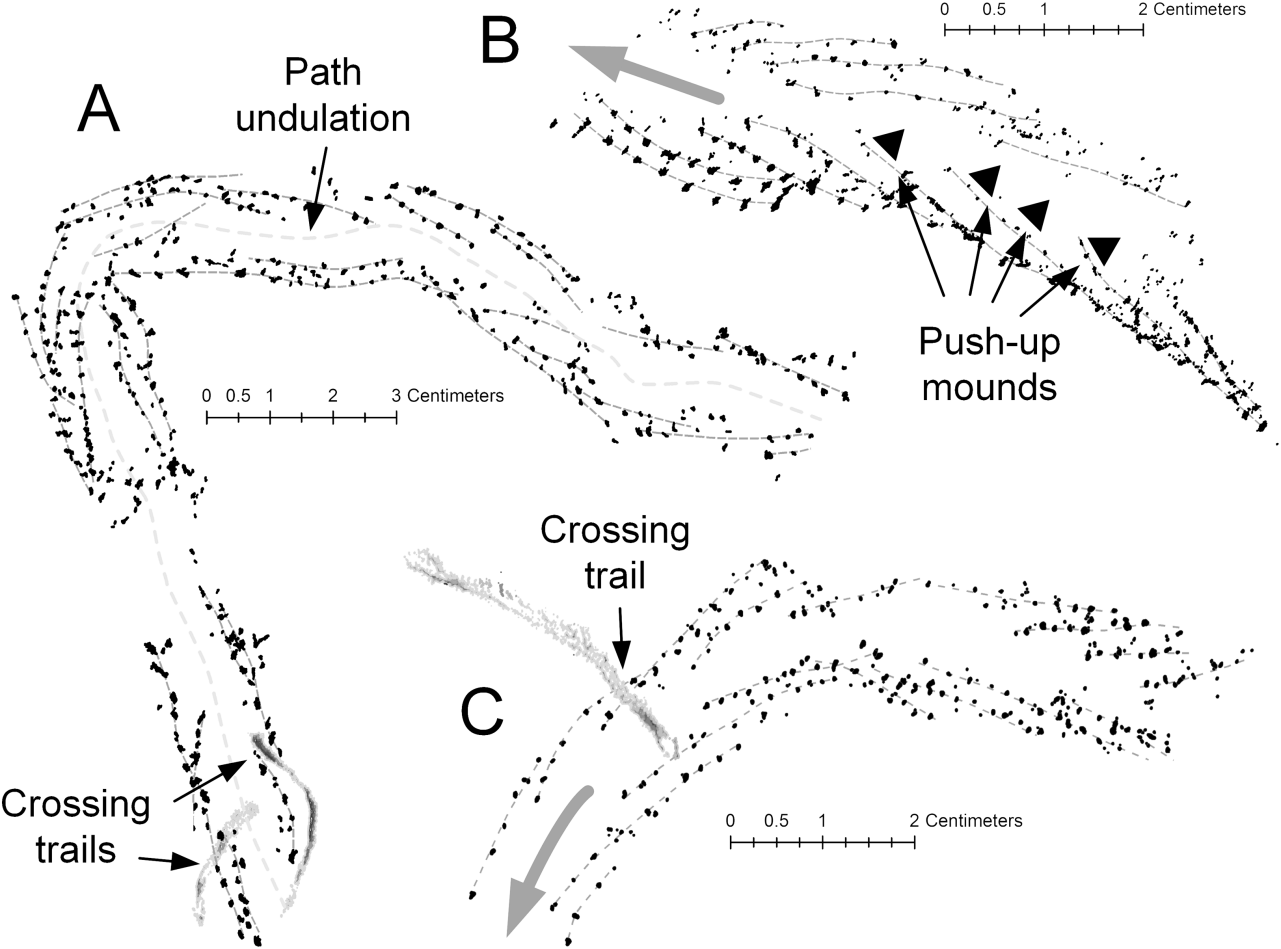
*Diplichnites gouldi* trackways, Hartselle Sandstone. Individual track series are marked with light gray dashed lines. (A) A curving track, showing the undulating midline and two crossing trails, MMNS IP-10062. (B) Same track as Fig. 10D, moving up a ripple face. Black triangles indicate push-up mounds opposite animal movement. En-echelon track rows indicate direction of travel (gray arrow), MMNS IP-10025. (C) Same track as Fig. 11D. Curving track with crossing trail and variation in gait. En-echelon track rows indicate direction of travel (gray arrow), MMNS IP-10018.

The traces of *D. gouldi* comprise a double row of parallel tracks lacking any evidence of a medial impression. In better-preserved specimens (Fig. 13), the tracks can be resolved into sets of pits aligned in staggered, oblique, en-echelon rows. Minter, Mángano & Caron (2012) ascribe similar en-echelon trackways of Cambrian trilobites to *Diplichnites*, but the Hartselle trackways are identical to those described by Manton (1952) for centipedes. The stride, measured as the length of a single track row (Minter, Braddy & Davis, 2007), varies from as short as five to six steps with row overlap of two to three steps, indicating a slow gait (Fig. 13A), to groups of 9–10 individual steps with overlap of five to six steps, indicating a faster gait (Fig. 13B). The pattern, where clearly preserved, indicates the direction of travel of the animal (Manton, 1952). Long trackways of *D. gouldi* (Fig. 13A) also demonstrate undulatory movement, characteristic of centipedes when moving quickly (Aoi, Egi & Tsuchiya, 2013) but not of trilobites or other marine arthropods. The trackways have a maximum width of 15–18 mm, which is within the range seen in modern centipedes. The track rows often appear to overlap in double sets, which suggests 11–19 pairs of legs and an overall length of less than 10 cm.

The detailed preservation of the tracks of *D. gouldi* themselves provide evidence of a partially saturated sediment surface exposed to the air. The response of arthropod tracks to water saturation of the substrate was described by McNamara (2014) for Paleozoic arthropod trackways. There are several Hartselle examples in which tracks of *D. gouldi*, on a single bedding plane, appear to move from saturated to unsaturated sand (Fig. 11C), a common feature on the modern flats where water saturation is highest in the troughs of ripples and tracks become indistinct. There are also cases where tracks move upslope and appear to slip in loose sand (Figs. 10D and 13B), leaving push-up mounds opposite the direction of movement, like those described for *Diplichnites* isp. by McNamara (2014). Tracks attributed to *Uchirites* by Rindsberg (1994), morphologically similar to the tracks of *Chiridotea coeca* and other isopod tracks (Uchman & Pervesler, 2006), often terminate when crossed by *D. gouldi* (Fig. 11D). In these cases, the stride lengths of *D. gouldi* vary, indicating that the animal turned toward and slowed before the encounter (Figs. 13A and 13C). Perhaps most intriguing are the congregating tracks of multiple *D. gouldi* that lead to chaotic “feeding frenzy” traces (Fig. 11E) suggestive of opportunistic scavenging and intraspecific competition for food, a behavior not unlike that shown by the modern gull *Larus argentatus smithsonianus* (Fig. 7F).

The prevalence of *D. gouldi* tracks and their interpretation as feeding/scavenging traces of a littoral chilopod are evidence for connection with an extensive terrestrial environment adjacent to the tidal flat. The *D. gouldi* tracks also provide evidence for both the nearshore location of the associated ichnocoenosis and a time period of exposure sufficient for this type of feeding behavior. A lengthy exposure was particularly important for the chilopod trace makers, which could rely only on terrestrial locomotion to avoid the incoming tide, unlike modern avian apex predators. These considerations make the evidence for surface predation and scavenging an important factor for recognition of a meso- to macro-tidal littoral environment.

## Conclusions

Many of the ethological classes (Seilacher, 1964), pascichnia (grazing traces), repichnia (locomotory traces), many of the domichnia (dwelling traces), and likely some digestichnia (digestive traces) (Vallon, 2012), found in the Hartselle can be attributed to crustacean and myriapod arthropods rather than trilobites or worms. These traces were made on exposed intertidal sediment and the data reported in this study support an interpretation of meso- to macro-tidal deposition.

This study defines criteria to recognize tidal flat deposits in a meso- to macro-tidal regime where waves and freshwater influx are minimal or non-existent. These criteria include (1) limited influence of wind and waves on the depositional environment; (2) lack of significant freshwater influence and therefore any persistent brackish environments; (3) a distinct spatial distribution of microenvironments defined by substrate and exposure period; (4) high diversity of epichnial traces directly associated with microenvironments across the tidal flat; (5) generally low degree of reworking of traces by bioturbation but high degree of reworking by tidal currents; and (6) preservation of traces of predation and scavenging behavior on an exposed surface.

The Hartselle Sandstone study section is comparably characterized by (1) marine body fossils in the lower described unit, indicative of a sublittoral environment distal from the shoreline; (2) wavy and flaser bedding and crossbedding in the higher described units demonstrative of tidal influence; (3) the vertical distribution of microenvironments representing a regressive, distal to proximal setting; (4) low degree of reworking of traces or bedding by subsequent bioturbation; (5) high diversity of epichnial traces; and (6) traces interpreted as recording predation and scavenging on an exposed surface. These features, together with the regional depositional pattern of the Hartselle Sandstone as tide-influenced bars and shoals, support a meso- to macro-tidal interpretation of the depositional environment.

It seems difficult to imagine how such a variety of epichnial traces could be preserved so well in an environment with the periodic inundation and exposure of a tidal flat. Experiments have shown that the shear strength of modern Bay of Fundy tidal flat sediments is increased by more than an order of magnitude when exposed mid-day in the summer when evaporation is high (Amos, Van Wagoner & Daborn, 1988) and erodibility of the sediment surface decreases inversely (Amos et al., 1992). Studies have also shown that damp sand is stable across a range of water saturations (Scheel et al., 2008). The presence of biofilms is a characteristic of the modern Lubec tidal flat and the formation of a sun-dried biofilm could serve to bind the sediment (Grant, Mills & Hopper, 1986) and such preservation may be evidence for biofilms. In a fetch-limited, non-estuarine setting, such a mechanism could be sufficient to preserve detailed epichnial traces (Seilacher, 2008).

## Supplemental Information

10.7717/peerj.6975/supp-1 Supplemental Information 1 Bulk sediment sample sieve analyses from modern tidal flat, Lubec, Maine. Sample numbers correspond to samples in Table S1. Gross, tare, and net total sample weights in grams are in blue. Sieve data are shown in purple. Sieve No. is ASTM US Sieve Number, Sieve Size is mesh opening in mm/in, Phi is mesh opening in ø, Tare, Gross, and Net weights for each sieve are in grams, with conversions to percent retained and cumulative percents.

10.7717/peerj.6975/supp-2 Supplemental Information 2 Bulk sediment sample locations, in UTM and Longitude–Latitude coordinates, with elevations. Lubec, Maine study area. Sample numbers are for reference. Easting and northing units are meters corresponding to Universal Transverse Mercator (UTM) Zone 19 North projection; datum is North American Datum 1983 (NAD83). Longitude is longitude west of the Greenwich meridian, Latitude is latitude, both are in degrees. GPS_Elevation is elevation in meters measured by GPS. Calibrated is elevation in meters, corrected using local sea level and tidal data published by the National Oceanic and Atmospheric Administration (NOAA). SedGroup corresponds to one of three grain-size categories: 1 = Gravel and coarse sand, 2 = Medium sand, 3 = Fine sand and silt.

10.7717/peerj.6975/supp-3 Supplemental Information 3 Talitrid and hyalid amphipod crustaceans exposed under sea wrack, upper shoreface facies, Lubec, ME. Video captured by Louis G. Zachos, August 2, 2016.

10.7717/peerj.6975/supp-4 Supplemental Information 4 Isopod crustacean * Chiridotea coeca* burrowing into substrate, *Chiridotea* ichnocoenosis, Lubec, ME. Video captured by Louis G. Zachos, July 3, 2017.

10.7717/peerj.6975/supp-5 Supplemental Information 5 Polychaete annelid *Nereis* sp. moving through biofilm, * Chiridotea* ichnocoenosis, Lubec, ME. Video captured by Louis G. Zachos, August 3, 2018.

10.7717/peerj.6975/supp-6 Supplemental Information 6 Amphipod crustacean *Corophium volutator* in its U-shaped burrow, *Corophium* ichnocoenosis, Lubec, ME. Video captured by Louis G. Zachos, August 11, 2016.

## Additional Information and Declarations

### Data Availability

The following information was supplied regarding data availability: Bulk sediment sample sieve analyses and GPS locations of these samples are available in Table S1 and Table S2, respectively. Four video clips of trace making organisms are provided as Video S1, Video S2, Video S3, and Video S4.

## References

[ref-1] Amos CL, Gordon DC, Dadswell MJ (1984). An overview of sedimentological research in the Bay of Fundy. Update on the Marine Environmental Consequences of Tidal Power Development in the Upper Reaches of the Bay of Fundy.

[ref-2] Amos CL, Daborn GR, Christian HA, Atkinson A, Robertson A (1992). In situ erosion measurements on fine-grained sediments from the Bay of Fundy. Marine Geology.

[ref-3] Amos CL, Van Wagoner NA, Daborn GR (1988). The influence of subaerial exposure on the bulk properties of fine-grained intertidal sediment from Minas Basin, Bay of Fundy. Estuarine, Coastal and Shelf Science.

[ref-4] Anthony EJ, Orford JD (2002). Between wave- and tide-dominated coasts: the middle ground revisited. Journal of Coastal Research.

[ref-5] Aoi S, Egi Y, Tsuchiya K (2013). Instability-based mechanism for body undulations in centipede locomotion. Physical Review E.

[ref-6] Barber AD (2009). Littoral myriapods: a review. Soil Organisms.

[ref-7] Barber AD (2011). Geophilomorph centipedes and the littoral habitat. Terrestrial Arthropod Reviews.

[ref-8] Barber AD (2019). World database of littoral myriapoda. http://www.marinespecies.org/myriapoda.

[ref-9] Baucon A, Felletti F (2013). Neoichnology of a barrier-island system: the Mula di Muggia (Grado lagoon, Italy). Palaeogeography, Palaeoclimatology, Palaeoecology.

[ref-10] Behbehani MI, Croker RA (1982). Ecology of beach wrack in northern New England with special reference to *Orchestia platensis*. Estuarine, Coastal and Shelf Science.

[ref-11] Benson KR (2002). The study of vertical zonation on rocky intertidal shores—a historical perspective. Integrative and Comparative Biology.

[ref-12] Brady F (1943). The distribution of the fauna of some intertidal sands and muds on the Northumberland coast. Journal of Animal Ecology.

[ref-13] Brafield AE, Newell GE (1961). The behaviour of *Macoma balthica* (L.). Journal of the Marine Biological Association of the United Kingdom.

[ref-14] Buatois LA, Gingras MK, MacEachern J, Mángano MG, Zonneveld J-P, Pemberton SG, Netto RG, Martin AJ (2005). Colonization of brackish-water systems through time: evidence from the trace-fossil record. Palaios.

[ref-15] Buatois LA, Mángano MG (2011). Ichnology: organism-substrate interactions in space and time.

[ref-16] Buatois LA, Wisshak M, Wilson MA, Mángano MG (2017). Categories of architectural designs in trace fossils: a measure of ichnodisparity. Earth-Science Reviews.

[ref-17] Christian JR, Grant CGJ, Meade JD, Noble LD (2010). Habitat requirements and life history characteristics of selected marine invertebrate species occurring in the newfoundland and labrador region.

[ref-18] Colombini I, Chelazzi L (2003). Influence of marine allochthonous input on sandy beach communities. Oceanography and Marine Biology: An Annual Review.

[ref-19] Dahl E (1952). Some aspects of the ecology and zonation of the fauna on sandy beaches. Oikos.

[ref-20] Dalrymple RW (1984). Morphology and internal structure of sandwaves in the Bay of Fundy. Sedimentology.

[ref-21] Dashtgard SE, Gingras MK (2005). Facies architecture and ichnology of recent salt-marsh deposits: Waterside Marsh, New Brunswick, Canada. Journal of Sedimentary Research.

[ref-22] Dashtgard SE, Gingras MK, Pemberton SG (2008). Grain-size controls on the occurrence of bioturbation. Palaeogeography, Palaeoclimatology, Palaeoecology.

[ref-23] Dawson JW (1873). Impressions and footprints of aquatic animals and imitative markings on Carboniferous rocks. American Journal of Science.

[ref-24] Ellis JC, Chen W, O’Keefe B, Shulman MJ, Witman JD (2005). Predation by gulls on crabs in rocky intertidal and shallow subtidal zones of the Gulf of Maine. Journal of Experimental Marine Biology and Ecology.

[ref-25] Elmhirst R (1932). XXI.—Studies in the Scottish marine fauna.—The crustacea of the sandy and muddy areas of the tidal zone. Proceedings of the Royal Society of Edinburgh.

[ref-26] Featherstone RP, Risk MJ (1977). Effect of tube-building polychaetes on intertidal sediments of the Minas Basin, Bay of Fundy. Journal of Sedimentary Petrology.

[ref-27] Feldmann RM (1989). Whitening fossils for photographic purposes. Paleontological Society Special Publications.

[ref-28] Folk RL (1980). Petrology of sedimentary rocks.

[ref-29] Forbes MR, Boates JS, McNeil NL, Brison AE (1996). Mate searching by males of the intertidal amphipod *Corophium volutator* (Pallas). Canadian Journal of Zoology.

[ref-30] Frey RW, Howard JD, Hong J-S (1987). Prevalent lebensspuren on a modern macrotidal flat, Inchon, Korea: ethological and environmental significance. Palaios.

[ref-31] Gates O, Anderson WA, Borns HW (1989). The Geology and Geophysics of the Passamaquoddy Bay Area, Maine and New Brunswick, and their Bearing on Local Subsidence. Neotectonics of Maine; Studies in Seismicity, Crustal Warping, and Sea-Level Change.

[ref-32] Getty PR, Bush AM (2017). On the ichnotaxonomic status of *Haplotichnus indianensis* (Miller, 1889). Ichnos.

[ref-33] Getty PR, Sproule R, Stimson MR, Lyons PC (2017). Invertebrate trace fossils from the Pennsylvanian Rhode Island Formation of Massachusetts, USA. Atlantic Geology.

[ref-34] Gingras MK, MacEachern JA, Dashtgard SE (2012). The potential of trace fossils as tidal indicators in bays and estuaries. Sedimentary Geology.

[ref-35] Grant J, Mills EL, Hopper CM (1986). A chlorophyll budget of the sediment-water interface and the effect of stabilizing biofilms on particle fluxes. Ophelia.

[ref-36] Griffith H, Telford M (1985). Morphological adaptations to burrowing in *Chiridotea coeca* (Crustacea, Isopoda). Biological Bulletin.

[ref-37] Hamilton DJ, Diamond AW, Wells PG (2006). Shorebirds, snails, and the amphipod (*Corophium volutator*) in the upper Bay of Fundy: top–down vs. bottom–up factors, and the influence of compensatory interactions on mudflat ecology. Hydrobiologia.

[ref-38] Häntzschel W (1939). Die Lebensspuren von *Corophium volutator* (Pallas) und ihre paläontologische Bedeutung. Senckenbergiana.

[ref-39] Hauck TE, Dashtgard SE, Gingras MK (2008). Relationships between organic carbon and pascichnia morphology in intertidal deposits: Bay of Fundy, New Brunswick, Canada. Palaios.

[ref-40] Hauck TE, Dashtgard SE, Pemberton SG, Gingras MK (2009). Brackish-water ichnological trends in a microtidal barrier island-embayment system, Kouchibouguac National Park, New Brunswick, Canada. Palaios.

[ref-41] Hertweck G, Wehrmann A, Liebezeit G, Steffens M (2005). Ichnofabric zonation in modern tidal flats: palaeoenvironmental and palaeotrophic implications. Senckenbergiana Maritima.

[ref-42] Jensen KT, André C (1993). Field and laboratory experiments on interactions among an infaunal polychaete, *Nereis diversicolor*, and two amphipods, *Corophium volutator & C. arenarium*: effects on survival, recruitment and migration. Journal of Experimental Marine Biology and Ecology.

[ref-43] Johannesson K, Saltin SH, Duranovic I, Havenhand JN, Jonsson PR (2010). Indiscriminate males: mating behaviour of a marine snail compromised by a sexual conflict?. PLOS ONE.

[ref-44] Kaplan MR (2007). Major ice sheet response in eastern New England to a cold North Atlantic region, ca. 16–15 cal ka BP. Quaternary Research.

[ref-45] Kelley JT, Belknap DF, Walsh JA, Randazzo G, Jackson D, Cooper A (2015). Tidal flat-barrier spit interactions in a fetch-limited, macro-tidal embayment, Lubec, Maine, USA. Sand and Gravel Spits.

[ref-46] Klein GDV (1963). Bay of Fundy intertidal zone sediments. Journal of Sedimentary Research.

[ref-47] Kopaska-Merkel DC, Rindsberg AK (2016). Bioirrigation in *Alph* n. igen., arthropod cubichnia from the Mississippian Hartselle Sandstone of Alabama (USA). Geodinamica Acta.

[ref-48] Lacefield J (2013). Lost Worlds in Alabama Rocks.

[ref-49] Larsen PF, Gilfillan ES (2004). A preliminary survey of the subtidal macrobenthic invertebrates of Cobscook Bay, Maine. Northeastern Naturalist.

[ref-50] Lee FT, Diehl SF, Anderson WA, Borns HW (1989). Geomechanical aspects of subsidence in eastern Maine. Neotectonics of Maine; Studies in Seismicity, Crustal Warping, and Sea-Level Change.

[ref-51] Lyell C (1845). Travels in North America, in the years 1841-2; with geological observations on the United States, Canada, and Nova Scotia.

[ref-52] MacMillan MR, Quijón PA (2012). Wrack patches and their influence on upper-shore macrofaunal abundance in an Atlantic Canada sandy beach system. Journal of Sea Research.

[ref-53] Mángano MG, Buatois LA (2004). Ichnology of Carboniferous tide-influenced environments and tidal flat variability in the North American Midcontinent. Geological Society of London Special Publications.

[ref-54] Mángano MG, Buatois LA, West RR, Maples CG (2002). Ichnology of a Pennsylvanian Equatorial Tidal Flat–The Stull Shale Member at Waverly, Eastern Kansas.

[ref-55] Manton SM (1952). The evolution of arthropodial locomotory mechanisms-Part 3. The locomotion of the Chilopoda and Pauropoda. Zoological Journal of the Linnean Society.

[ref-56] Martinsson A, Crimes TP, Harper JC (1970). Toponomy of trace fossils. Trace Fossils: Geological Journal Special Issue 3.

[ref-57] McDermott JJ (2005). Food habits of the surf-zone isopod *Chiridotea caeca* (Say, 1818) (Chaetiliidae) along the coast of New Jersey, U.S.A. Proceedings of the Biological Society of Washington.

[ref-58] McLachlan A, McLachlan A, Erasmus T (1983). Sandy beach ecology: a review. Sandy Beaches as Ecosystems.

[ref-59] McNamara KJ (2014). Early Paleozoic colonisation of the land: evidence from the Tumblagooda Sandstone, Southern Carnarvon Basin, Western Australia. Journal of the Royal Society of Western Australia.

[ref-60] Meadows PS (1964). Substrate selection by *Corophium* species: the particle size of substrates. Journal of Animal Ecology.

[ref-61] Meadows PS, Reid A (1966). The behaviour of *Corophium volutator* (Crustacea: Amphipoda). Journal of Zoology.

[ref-62] Minter NJ, Braddy SJ (2009). Ichnology of an early Permian intertidal flat: the Robledo Mountain Formation of southern New Mexico, USA. Special Papers in Palaeontology.

[ref-63] Minter NJ, Braddy SJ, Davis RB (2007). Between a rock and a hard place: arthropod trackways and ichnotaxonomy. Lethaia.

[ref-64] Minter NJ, Mángano MG, Caron J-B (2012). Skimming the surface with Burgess Shale arthropod locomotion. Proceedings of the Royal Society B: Biological Sciences.

[ref-65] Newcombe CL (1935a). A study of the community relationships of the sea mussel, *Mytilus edulis* L. Ecology.

[ref-66] Newcombe CL (1935b). Certain environmental factors of a sand beach in the St Andrews Region, New Brunswick, with a preliminary designation of the intertidal communities. Journal of Ecology.

[ref-67] NOAA (2014). Digital coast data access viewer. https://coast.noaa.gov/dataviewer.

[ref-68] Oldale RN, Tucker RD, Marvinney RG (1989). Timing and mechanisms for the deposition of the glaciomarine mud in and around the Gulf of Maine; a discussion of alternative models. Studies in Maine Geology.

[ref-69] Pashin JC, Gastaldo RA, Greb SF, Chesnut DR (2009). Carboniferous of the Black Warrior Basin. Carboniferous Geology and Biostratigraphy of the Appalachian Basin.

[ref-70] Plante CJ (2010). Landscape and smaller-scale effects of lugworm (*Arenicola marina*) deposit feeding on benthic bacterial assemblages. Journal of Marine Research.

[ref-71] Pollock LW (1998). A Practical Guide to the Marine Animals of Northeastern North America.

[ref-72] Reise K (1977). Predator exclusion experiments in an intertidal mud flat. Helgoländer Wissenschaftliche Meeresuntersuchungen.

[ref-73] Rindsberg AK (1994). Ichnology of the upper Mississippian Hartselle Sandstone of Alabama, with notes on other Carboniferous formations.

[ref-74] Rindsberg AK, Kopaska-Merkel DC, Buta RJ, Rindsberg AK, Kopaska-Merkel DC (2005). *Treptichnus* and *Arenicolites* from the Steven C. Minkin Paleozoic Footptint Site (Langsettian, Alabama, USA). Pennsylvanian Footprints in the Black Warrior Basin of Alabama.

[ref-75] Sass DB (1962). Improved techniques for the photographing of fossils. Journal of Paleontology.

[ref-76] Scheel M, Seemann R, Brinkmann M, Di Michiel M, Sheppard A, Breidenbach B, Herminghaus S (2008). Morphological clues to wet granular pile stability. Nature Materials.

[ref-77] Seilacher A (1964). Sedimentological classification and nomenclature of trace fossils. Sedimentology.

[ref-78] Seilacher A (2008). Biomats, biofilms, and bioglue as preservational agents for arthropod trackways. Palaeogeography, Palaeoclimatology, Palaeoecology.

[ref-79] Shchepetkina A, Gingras MK, Zonneveld J-P, Pemberton SG (2016). Sedimentary fabrics of the macrotidal, mud-dominated, inner estuary to fluvio-tidal transition zone, Petitcodiac River estuary, New Brunswick, Canada. Sedimentary Geology.

[ref-80] Smith A, Braddy SJ, Marriott SB, Briggs DEG (2003). Arthropod trackways from the Early Devonian of South Wales: a functional analysis of producers and their behaviour. Geological Magazine.

[ref-81] Stenton-Dozey J (1983). Stranded kelp: its fauna and influence on sandy beach energetics MSc thesis.

[ref-82] Thomas WA (1972). Mississippian Stratigraphy of Alabama.

[ref-83] Thomas WA, Briggs G (1974). Converging clastic wedges in the Mississippian of Alabama. Carboniferous of the Southeastern United States.

[ref-84] Thomas WA, Mack GH (1982). Paleogeographic relationship of a Mississippian barrier-island and shelf-bar system (Hartselle Sandstone) in Alabama to the Appalachian-Ouachita orogenic belt. Geological Society of America Bulletin.

[ref-85] Thomas RG, Smith DG, Wood JM, Visser J, Calverley-Range EA, Koster EH (1987). Inclined heterolithic stratification—terminology, description, interpretation and significance. Sedimentary Geology.

[ref-86] Trewin NH (1995). A draft system for the identification and description of arthropod trackways. Palaeontology.

[ref-87] Trott TJ (2004). Cobscook Bay inventory: a historical checklist of marine invertebrates spanning 162 years. Northeastern Naturalist.

[ref-88] Tyler DA, Ladd JW (1980). Vertical crustal movement in Maine.

[ref-89] Uchman A, Pervesler P (2006). Surface lebensspuren produced by amphipods and isopods (crustaceans) from the Isonzo Delta tidal flat, Italy. Palaios.

[ref-90] Vallon LH, Hunt AP, Milàn J, Lucas SG, Spielmann JA (2012). *Digestichnia* (Vialov, 1972)—An almost forgotten ethological class for trace fossils. Vertebrate Coprolites.

[ref-91] Walsh JA (1988). Sedimentology and late Holocene evolution of the Lubec Embayment MSc thesis.

[ref-92] Warren JH (1990). Role of burrows as refuges from sub-tidal predators of temperate mangrove crabs. Marine Ecology Progress Series.

[ref-93] Watling L, Fegley J, Moring J (2003). Life Between the Tides, Marine Plants and Animals of the Northeast.

[ref-94] Wells MR, Allison PA, Piggott MD, Gorman GJ, Hampson GJ, Pain CC, Fang F (2007). Numerical modeling of tides in the Late Pennsylvanian midcontinent seaway of North America with implications for hydrography and sedimentation. Journal of Sedimentary Research.

[ref-95] Whitlach RB (1982). The ecology of New England tidal flats: a community profile.

[ref-96] Wilhelmsen U, Reise K (1994). Grazing on green algae by the periwinkle *Littorina littorea* in the Wadden Sea. Helgoländer Meeresuntersuchungen.

[ref-97] Williams GE (2000). Geological constraints on the Precambrian history of Earth’s rotation and the Moon’s orbit. Reviews of Geophysics.

[ref-98] Wilson GV (1987). Characteristics and resource evaluation of the asphalt and bitumen deposits of Northern Alabama.

